# Development of KASP markers, SNP fingerprinting and population genetic analysis of *Cymbidium ensifolium* (L.) Sw. germplasm resources in China

**DOI:** 10.3389/fpls.2024.1460603

**Published:** 2025-01-08

**Authors:** Baoming Shen, Airong Shen, Yun Tan, Lina Liu, Sainan Li, Zhuming Tan

**Affiliations:** Institute of Biodiversity, Hunan Academy of Forestry, Changsha, China

**Keywords:** *Cymbidium ensifolium*, SNP, KASP, genetic diversity, fingerprint

## Abstract

*Cymbidium ensifolium* (L.) Sw. is a valuable ornamental plant in the genus *Cymbidium*, family Orchidaceae, with high economic and ecological significance. However, the lack of population genetic information and molecular markers has hindered the development of the sales market and genetic breeding of *C. ensifolium* despite the abundance of commercial cultivars available. In this study, we aimed to develop a set of single nucleotide polymorphism (SNP) markers to distinguish the main cultivated *C. ensifolium* cultivars in China and provide technical support for domestic cultivar protection, registration, and market rights protection. A total of 1,280,516 high-quality loci were identified from 10,021,591 SNPs obtained by sequencing 50 C*. ensifolium* commercial cultivars using double digest restriction site-assisted DNA sequencing technology. A total of 7,599 SNPs were selected for kompetitive allele-specific PCR (KASP) primer design, and 4,360 were successfully designed as KASP markers. Population structure analysis revealed that the 50 commercial cultivars were best divided into four populations, with some correlation between the group distribution and the morphological and geographical characteristics of the germplasm. Using the genotyping results from 28 KASP markers screened from the cultivars, a minimum set of 11 markers was identified that could distinguish 83 C*. ensifolium* commercial cultivars completely, with the remaining 17 markers serving as extended markers. The average PIC value of the 11 markers was 0.345, which was considered medium polymorphism. DNA fingerprints were constructed for the 83 cultivars on the basis of the 11 KASP markers, providing a new approach for mapping DNA fingerprints in *C. ensifolium* cultivars with high efficiency, accuracy, and low cost compared with traditional methods.

## Introduction

*Cymbidium ensifolium* (L.) Sw. is a perennial evergreen herbaceous plant in the Orchidaceae family. *C. ensifolium* has a karyotype of 2N = 2X = 40 with chromosomes of different lengths and the genome size is 3.62 Gb (Ai et al., 2021; Li et al., 2002). It is an important species in traditional Chinese *cymbidium* due to its abundant flower colors, diverse flower types, diverse leaf colorations and unique historical and cultural significance (Cao et al., 2022). *C. ensifolium* has significant ornamental, cultural, medicinal and economic value (Jimoh et al., 2022; Yang and Zhu, 2015). There is a wide variety of *C. ensifolium*, with significant price differences among different varieties (Huang, 2012). However, since *C. ensifolium* varieties are distinguished mainly by flower type and color and some varieties have minimal differences, identification becomes challenging, especially during nonflowering periods (Chen et al., 2022). Traditional morphological identification methods are often insufficient, leading to inaccurate identification and causing discrepancies in orchid trading. This situation adversely affects the robust and orderly development of the orchid market. Additionally, the lack of accurate and reliable identification methods has resulted in confusion between identical *C. ensifolium* varieties with different names and different varieties with the same name, which hampers the identification, conservation, improvement, cultivation, and utilization of *C. ensifolium* genetic resources (Huang, 2012). Therefore, there is an urgent need to establish a simple, stable, and reliable identification method for *C. ensifolium* varieties.

DNA molecular markers have advantages such as short cycle times, minimal environmental influence, and high-throughput detection, providing new means for variety identification (Ramesh et al., 2020). Currently, several molecular marker technologies, including Random Amplified Polymorphic DNA (RAPD), which is based on ITS and cpDNA fragments, Inter-Simple Sequence repeat (ISSR), Simple Sequence Repeat (SSR) markers, and fluorescent SSR markers, have been applied for resource identification of *C. ensifolium* varieties (Li et al., 2014; Jin, 2019; Li, 2014; Wang et al., 2021a; Ai et al., 2019; Hu et al., 2008). However, RAPD is a dominant marker that cannot distinguish between heterozygous and homozygous genotypes, limiting its use in genetic analysis and genetic map construction (Congiu et al., 2000). ITS and limited cpDNA fragment-based markers can only differentiate a small number of varieties because of the limited number of polymorphic sites (Sivu et al., 2022). SSR markers are limited in quantity and detection throughput, have higher detection costs, and require time-consuming and labor-intensive data interpretation, as well as subjective misjudgment of band patterns due to human factors, which restrict their use in a wider range of variety identification work (Zhang et al., 2022b).

Single nucleotide polymorphisms (SNPs) refer to DNA sequence polymorphisms caused by variations such as substitutions and inversions of individual bases in the genomic DNA sequence. SNPs have advantages such as large quantity, wide distribution, allelic dimorphism, and stable inheritance (Li et al., 2023a). These characteristics make SNP-based molecular markers the latest generation of markers. With the emergence of various high-throughput SNP detection platforms, they can effectively compensate for the technical limitations of SSR markers. The construction of a DNA fingerprint map based on SNP markers is highly important for variety specificity identification, assessment of genetic variations, authenticity verification, identification of seed purity, and other characteristics (Nguyen et al., 2020; Josia et al., 2021; Wang et al., 2021b, 2015; Su et al., 2019). This method has been designated as one of the recommended marker methods by the International Union for the Protection of New Varieties of Plants (UPOV) and the General Guidelines for DNA Identification of Plant Varieties Using DNA markers (NY/T 2594-2016) (Button, 2008). The development of SNP molecular markers can be based on different DNA sources, such as SNP markers developed on the basis of specific genes, EST-SNP markers developed on the basis of express sequence tag (EST) sequences, GSS-SNP markers developed on the basis of gene survey sequence (GSS), and genomic SNPs developed on the basis of whole-genome data (Addison et al., 2020; Wang et al., 2019, 2020; Ashwath et al., 2023). Among these, genomic SNPs provide the most accurate identification but are relatively expensive. With the development of sequencing technology and the popularization of next-generation sequencing (NGS), the cost of sequencing has greatly decreased (van Dijk et al., 2014). Using transcriptome sequencing (RNA-seq), restriction site-associated DNA sequencing (RAD-seq) and double digest restriction site-associated DNA sequencing (ddRAD-seq), more abundant SNP loci can be obtained (Wang et al., 2015; Baird et al., 2008; Magbanua et al., 2023). Among these, RAD-seq and ddRAD-seq can identify SNP loci with broader coverage than can RNA-seq.

There are also various methods for detecting SNP molecular markers, such as direct sequencing, TaqMan probe, amplification refractory mutation system pCR (ARMS-PCR; also known as allele-specific PCR), molecular beacon, high-resolution melting analysis (HRM) technology, cleaved amplified polymorphic sequence (CAPS), SNaPshot, kompetitive allele-specific PCR (KASP), gene chip, and mass spectrometry (Zhao et al., 2017; Kovalchuk and Arkhipova, 2023; Zongze et al., 2018; Gerasimova et al., 2013; Gomes et al., 2018; Amanullah et al., 2022; Pradhan et al., 2023; Guan et al., 2023; Franklin et al., 2020; Wang et al., 2016). Among these, KASP technology has the advantages of low cost, high throughput, time and labor savings, and convenience (He et al., 2014). It has become one of the main methods for SNP genotyping and insertion/deletion (InDel) detection internationally and has been successfully applied in genetic typing and variety breeding of grain crops and economic crops, such as wheat, rice, maize, strawberry, grape, broccoli, cotton, tobacco, and peach (Liu et al., 2023; Tang et al., 2022; Chen et al., 2021; Yang et al., 2020; Wang et al., 2022; Shen et al., 2021; Zhao et al., 2021; Wang et al., 2021b; Fleming et al., 2022). However, there are currently no reports on the application of SNP markers developed on the basis of KASP technology for variety identification, fingerprint map construction, and systematic classification in orchids such as *Cymbidium*.

Therefore, we utilized KASP technology to screen a set of SNP markers that can distinguish *C. ensifolium* germplasm resources in China. In this study, 50 C*. ensifolium* germplasm resources were subjected to Illumina NovaSeq sequencing (**Supplementary Table 1**). The results were compared with those of the reference genome to identify SNP core markers and interpret their genetic relationships, genetic diversity, and population structure. Additionally, a DNA fingerprint of 83 *C. ensifolium* varieties was created to effectively differentiate between different varieties of *C. ensifolium* (**Supplementary Table 2**). These results provide a scientific foundation and data reference for genetic diversity analysis, variety identification, and molecular breeding of *C. ensifolium*.

## Materials and methods

### Plant materials

**Figure 1:**
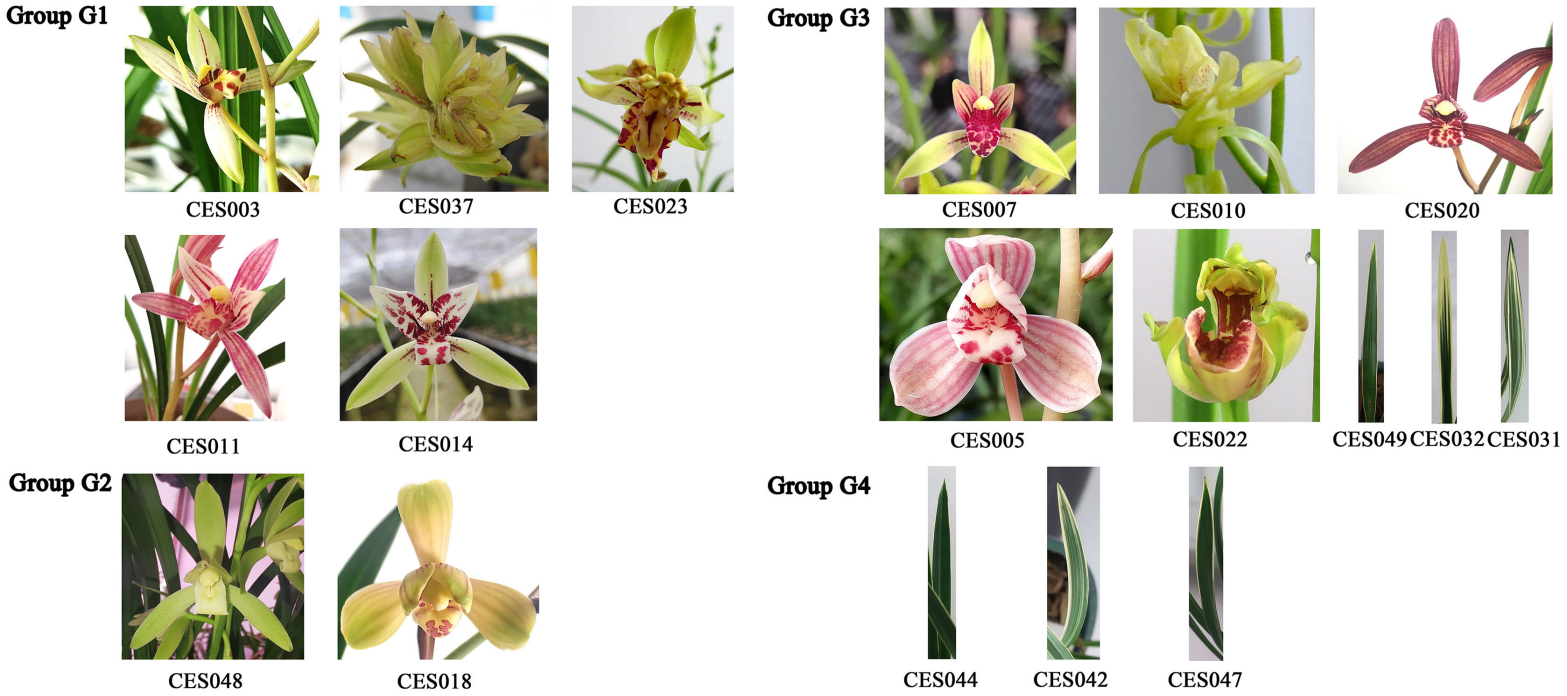
Representative flower and leaf characteristics of the tested *C. ensifolium* varieties.

Wild *C. ensifolium* resources are included in the National Key Protected Wild Plant List in China, and it is prohibited by law to collect them from the wild. This study adhered to all relevant institutional, national, and international guidelines and laws. No prior permission was required to conduct research on this species. All the plants used in this study are well-known commercial cultivars in China cultivated in pots in a greenhouse under controlled temperature and lighting conditions, a greenhouse on day/night temperatures of 30/23°C under a 14 h light/10 h dark photoperiod. The plant material used in this study was identified by Prof. Zhuming Tan (see author list) and stored at the laboratory of the Hunan Academy of Forestry. To encompass more genetic diversity and obtain a broader range of SNP markers, the selected materials represented diverse morphological variations of *C. ensifolium*, including leaf, sepal, lip, and petal color, size and shape, leaf color and shape, sepal size and shape, lip size and shape, and petal size and shape (**Figure 1**). Five vigorous plants were randomly selected for each germplasm resource, and young leaf samples were collected, frozen at -80°C, and stored for DNA extraction.

For the preliminary screening of KASP markers, 50 representative *C. ensifolium* cultivars were used (**Supplementary Table 1**). The rescreening and fingerprint construction using KASP markers included 83 *C. ensifolium* cultivars (**Supplementary Table 2**).

### DNA extraction, library construction and simplified genome sequencing

After the leaf samples were ground, genomic DNA was extracted using a D312 Universal Plant DNA Kit (Genepioneer, China), and the quality and concentration of the DNA were measured, and the purity (OD_260 nm_/OD_280 nm_=1.8–2.0) was determined using a NanoDrop2000 UV spectrophotometer (Thermo Fisher, Waltham, MA, USA). A paired-end library with a length range of 300-500 bp was subsequently constructed using digest restriction site-associated DNA sequencing library construction of qualified sample DNA (Peterson et al., 2012). First, 500 ng of genomic DNA was incubated with 0.6 U EcoRI (NEB), T4 DNA ligase (NEB), ATP (NEB), and EcoRI connectors (including index sequences of differentiated samples) at 37°C for 3 h and annealed at 65°C for 1 h. The restriction enzyme NlaIII (NEB) and the NlaIII connector were then added and incubated for 3 h at 37°C. After the reaction, the endonuclease was inactivated at 65°C for 30 min in a polymerase chain reaction (PCR) amplifier. The 400–600 bp digested products were recovered using a MiniBEST Agarose Gel DNA Extraction Kit (Takara, China) and quantified using a NanoDrop2000 UV spectrophotometer (Thermo Fisher, Waltham, MA, USA). After 50 samples were mixed in equal quantities, the Illumina NovaSeq 6000 PE150 platform was sequenced and used to construct a DNA library of the mixed products.

### Raw data quality control

Raw data quality control was conducted on the original sequenced reads. Fastp software (version: 0.20.0, https://github.com/OpenGene/fastp) was used to eliminate reads with an unknown base number N<5, a quality value<5, connector sequences, and other low-quality sequences to obtain clean data. Burrows–Wheeler Aligner (BWA) (version: 0.7.17-R1188, https://github.com/lh3/bwa) was then used to align the sequenced reads with the reference genome (Li and Durbin, 2009). The reference genome used was the GWHBCII00000000 genome Fasta (*C. ensifolium*) (download website: https://ngdc.cncb.ac.cn/gwh/Assembly/20686/show), with parameters set as –M -R [51]. The insert size and coverage depth of each sample were determined, and variation was detected by comparing the positions of the clean reads in the reference genome (Li et al., 2023a). SAMtools software (version: v1.9, https://github.com/samtools/samtools) was used to statistically analyze the sequencing depth, genome coverage, insertion fragment length, and other information of each simplified genome sample (Li et al., 2009).

### SNP analysis

SNP analysis was primarily performed using the Genome Analysis Toolkit (GATK) software package (version: 4.1.4.1, https://github.com/broadinstitute/gatk). Using the positioning of clean reads in the reference genome, GATK was used to detect SNPs and obtain the final SNP site set, followed by SNP statistics (McKenna et al., 2010). The main detection process included the following steps: (1) Picard’s (version: 0.7.17-r1188, https://github.com/broadinstitute/gatk) Mark Duplicate tool was used to remove duplicates and mask the effects of PCR duplication on the results from BWA alignment; (2) variant calling was performed using GATK, including SNP and InDel; and (3) variant quality score recalibration (VQSR) was performed using GATK. (4) GATK was used to filter the obtained variants (filtering out sites with QD< 2.0, MQ< 40.0, FS > 60.0, SOR > 6.0, MQRankSum< -12.5, ReadPosRankSum< -8.0), selecting reliable variant results, followed by statistical analysis and bar chart plotting of the variant types by GATK. SNP site annotation was implemented with SnpEff (version: 4.3t, http://pcingola.github.io/SnpEff).

### Population SNP filtering

To obtain high-quality SNPs for population genetic analysis of *C. ensifolium*, a series of standards were applied for preliminary screening. These standards included criteria such as average sequencing depth ≥5×, minor allele frequency (MAF) ≥0.05, information integrity ≥0.70, SNP quality value Q ≥30, and number of alleles of 2 (Li et al., 2023a). The SNP density distribution across each chromosome was subsequently calculated and visualized. The average values of the population genetic indices in 50 *C. ensifolium* samples were also calculated and statistically analyzed.

### Genetic diversity and population structure analysis

Genetic diversity and population structure analyses were performed using GCTA software (version: 1.92.1, http://cnsgenomics.com/software/gcta/#Overview) for principal component analysis (PCA) on the basis of the high-quality SNPs obtained. The maximum likelihood (ML) method in RAxML software (version 8.2.12, https://github.com/stamatak/standard-RAxML/) was used to construct an evolutionary tree of 50 *C. ensifolium* samples. Admixture software (version: 1.3.0, http://software.genetics.ucla.edu/admixture/) was used to analyze the population genetic structure.

### SNP site filtering and KASP primer design

Due to the difficulty in distinguishing between samples in this study, a combination of conventional filtering and manual selection of difficult-to-distinguish varieties was used in the SNP site selection strategy. After considering both filtering criteria, the selection criteria for SNP sites used in KASP primer development in this study were defined as follows: first, conserve the sequence flanking the SNP site on the DNA chain of the chromosome for more than 50 bp; second, retain markers with an average depth of 5X or greater, with the SNP being a biallelic gene; third, trim 100 bp sequences upstream and downstream of the SNP marker, then use blast software (version: 2.10.1+, https://blast.ncbi.nlm.nih.gov/Blast.cgi) to align the sequence against the reference genome and remove markers with multiple alignment positions; fourth, retain markers with a polymorphic information content (PIC) greater than 0.020, (the low PIC threshold is due to manual selection considerations), and the PIC calculation formula refers to the method of Zhang, et al., where *Pi* and *Pj* are the frequency of occurrence of the two alleles of SNP in all varieties tested, and l is the number of samples (Zhang et al., 2020).


$$PIC=1-\sum_{\text{i}=0}^{\text{l}}{\text{P}}_{i}^{2}-\sum_{\text{i}=1}^{\text{l}-1}\sum_{\text{j}=\text{i}+1}^{\text{l}}2P_{\text{i}}^{2}P_{\text{j}}^{2}$$

For each selected SNP marker, primer design was conducted using Primer3 software (version: 2.4.0, https://sourceforge.net/projects/primer3/files/latest/download). Sequences 100 bp upstream and downstream of candidate SNPs were used for KASP marker design. Two allele-specific primers and one universal primer were designed for each KASP target site. The primer design parameters were set as follows: GC content<60%, melting temperature (Tm) between 57 and 63°C, and PCR product size not exceeding 120 bp. Further optimization of primer design results can be conducted on the basis of actual verification results. Only SNP sites with successful primer design were considered to be qualified KASP markers and were used for downstream analysis. The distribution statistics of the markers with successful primer design were also based on the annotation results.

### Verification of SNP locus authenticity and KASP genotyping

To save costs, 28 randomly selected SNP sites with successful KASP primer design were preliminarily screened using Perl scripts, with 5 samples selected for first-generation sequencing validation. The distribution of developed KASP markers on the chromosomal was visualized using Tbtools-II (version: 2.138) (Chen et al., 2023).

Primers were designed, conventional sequencing was performed, and the authenticity of the SNP site was verified (**Table 1**). For successfully validated sites, KASP primers were redesigned using the real first-generation sequencing results with Primer3 (version: 2.4.0, https://sourceforge.net/projects/primer3/files/latest/download). The primers were synthesized by Sangon Biotech (Shanghai) Co., Ltd., with FAM- or VIC-tails (FAM-tail: 5′-GAAGGTGACCAAGTTCATGCT-3′; VIC-tail: 5′- GAAGGTCGGAGTCAACGGATT -3′) (**Supplementary Excel 1**). Subsequently, KASP genotyping experiments were subsequently conducted.

**Table 1:** Primers used for the initial screening validation of candidate SNP loci.

Number	SNP Position	Forward primer (5′-3′)	Reverse primer (5′-3′)	Product length/bp
1	Chr1-55623523	AGCGTGGTGGGATTGAACTT	ACGAAAGCACGACGTGTACA	242
2	Chr1-73862970	GATTGTCCTATTCAAGTCTCATTAGA	ACCTTTCTTGGCTTGGCAAA	164
3	Chr1-171445320	ACCAAACTGTGAGGCAACCA	CCCTCGACCGGTAAGTTCAC	196
4	Chr10-25134624	CGGAGCAAACCTCATCACCT	GCAACACACCAGAGGTACCA	191
5	Chr12-53446226	CTCTCGAGGAGCTATTGCAG	GCCCACTAAAGTGCAAAGCA	495
6	Chr12-63867683	AGAAGCGATCAAGAACCGGG	AGCATGTCTCACACACTGGT	278
7	Chr12-75662664	TTAGATGGGCCAGGCTTGTG	GGTGCATGGTGAGTTCAGCT	547
8	Chr13-6407723	GGATGGCTCCTGCTTCAGTT	CCTAACTACGCCCTTCGCAA	364
9	Chr13-34339702	TCAGTTTCGGACGAAGGTCG	ATGTTTGTGCCACGCGTTTC	271
10	Chr16-43018260	CTATGGGCGTAGAAGTGGTGT	AGTGTCAACATTGCGGCAAC	367
11	Chr16-66004509	GGCGCATCCAAAGAACAAGG	CCTCTAATATGATCTTGAAGTGCACC	308
12	Chr18-11319799	CCAAGAACCCCTCATGTCCC	TGAGCCATGATAATTGCAAGGT	381
13	Chr18-27339793	TCGCTTATCAGAAGTGCCACA	TGCTGTTTACTGCCCATCCA	532
14	Chr19-8308837	TGGCAGAAACCTATAGGCCT	TTCCCTGCCAAGCTCAATGT	177
15	Chr2-12538713	ACGGTTGCTCGGCTTTTCTA	ACCATCCAAACCGTAAGCTTCT	329
16	Chr2-77710898	GTCCCAAAGACAAGTTCCCA	GGCTTGCTAATACGTGGCAC	221
17	Chr2-114570305	TGCAAGCAAGATGTCTCCGT	CCACGCACTTACAGGAGGTT	244
18	Chr2-140839794	CGGAGCTTCAATAGTTAACAACAGT	TCTTTCTGATGCAGGGCTGG	475
19	Chr20-7711682	TGGTTTCCACCTTGCCTGTT	CTGATGGCTCCTGGTGTGTT	208
20	Chr20-9861983	TGAATATACATTTCAACCATGGAGTT	TTTTCCCTTGCGAGACTTGC	237
21	Chr3-67201341	TCGCTCCTTCGCTTGTTCAT	CAAGCAGCCCTTGGTATTGA	549
22	Chr3-153114895	GCCAAGCCTGAGGTGTTAGA	TGTGAGTCAGCTTGCACAGT	190
23	Chr4-57124129	CTCCACCACCTGCATCCATT	TGGATATCCATATGAAGGTGTTGCA	440
24	Chr4-103337174	TCCTGGTTCCCTGATCCCAT	GATAATGGGGGCCGAGAAGG	471
25	Chr5-47997902	TGCATCCTAACAAGACTTGGCA	ACAGTCAGTTAGAGCGCAACA	500
26	Chr8-90715849	GAGTCAGGTTTCCAAACGCG	AACATGTCGCCGTACAACCT	335
27	Chr9-22416891	GGACTTCAAGGAACTGGAGGA	AGAAAGAAACCGGCGCAAAC	511
28	Chr9-24025081	ACCTTGTTGGCCGCCTTTTA	AAGCCGAGCTGCAATAAACC	431

Each SNP location information consists of the number of chromosomes where the SNP site is located plus the location of the corresponding chromosome.

The KASP assay was performed in a 5 μL PCR system/condition comprising 2.5 μL of 2X KASP Master mix (JasonGen, China), 1.25 μL of primer mixture, and 1.25 μL of DNA at a concentration of 10–20 ng/mL. The PCR program included 10 min at 95°C, 10 touchdown cycles of 95°C for 20 s and 61–55°C for 60 s (decreasing by 0.6°C per cycle), and 27 cycles of 95°C for 20 s and 55°C for 60 s. Following PCR, fluorescence data were read and analyzed via the CFX Connect TM Real-Time System (Bio-Rad, USA). If the genotyping results were unsatisfactory, PCR optimization was performed with an additional 3 cycles of 95°C for 20 s and 55°C for 60 s.

### Fingerprint construction

For the obtained SNP genotyping results, the optimal combination of markers was calculated for fingerprinting using a Perl program. The genotypes of the optimal combination of markers were heatmapped for fingerprinting, with each row representing one SNP locus and each column representing one sample. The genotypes were color-coded as follows: AA=green, AG=light pink, CC=yellow, CT=grew, GT=dark red, TT=blue, AC=pink, AT=orange, CG=light blue, and GG=purple; and no call genotypes were designated as NN=white.

## Results

### Simplified genome sequencing and reference genome alignment

The sequencing of 50 *C. ensifolium* samples on the Illumina NovaSeq 6000 PE150 yielded a total of 64.89 Gb of clean data, with an average sequencing data volume of 1.30 GB per sample. The Q30 scores ranged from more than 89.79% to 94.26%, with an average exceeding 92.76%. The average Q score was between 35.24 and 36.07, and the GC content ranged from 33.86% to 35.17% (**Supplementary Table 3**). The clean reads obtained were mapped to the reference genome, with an average mapping efficiency of 99.50%. The genome average cover depth was 6.6X, the genome coverage at 5X averaged 1.92%, and that at 10X averaged 0.95% (**Supplementary Table 4**).

### Selection, identification and annotation of high-quality SNPs

**Figure 2:**
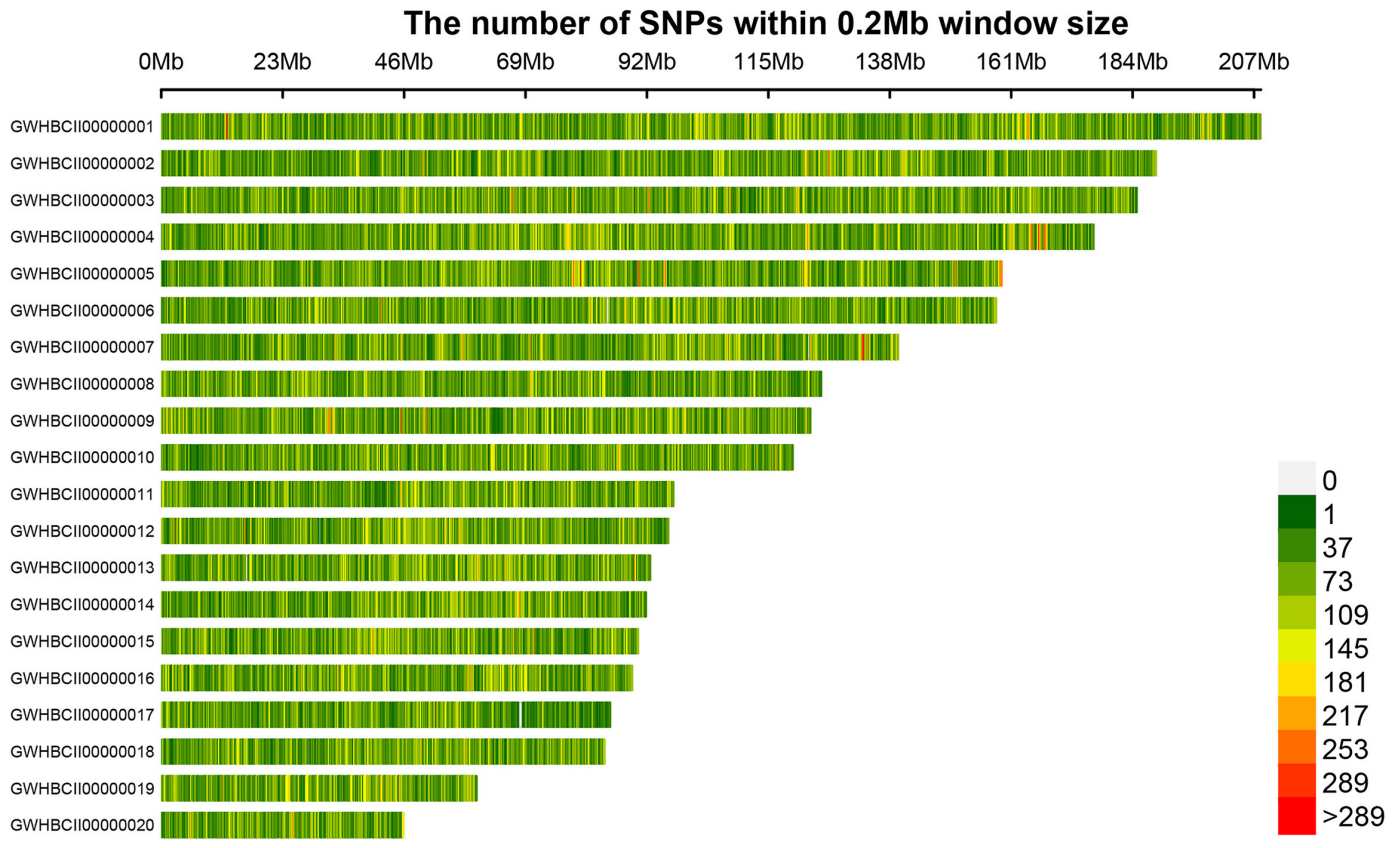
SNP density distribution on each chromosome. The horizontal axis represents the chromosome length, and the vertical axis represents the chromosome number. Different colors represent the number of SNPs in different regions.

Following sequencing, GATK software detected numerous SNP variants in the 50 *C. ensifolium* samples, resulting in a total of 10,021,591 SNPs, with each sample ranging from 691,307 to 1,612,362 (**Supplementary Table 5**). The most common types of SNP variations were C>T (2,034,818) and G>A (2,037,584), followed by A>G (1,289,885) and T>C (1,286,472), with the least common types being C>G (233,292) and G>C (233,361) (**Supplementary Figure 1A**, **Supplementary Excel 3**). A total of 1,280,516 filtered SNPs distributed across 20 main chromosomes were obtained for population genetic analysis (**Supplementary Figure 1B**, **Table 2**). An SNP distribution map was then created for the 20 main chromosomes on the basis of the number and density of SNPs (**Figure 2**). Chromosome GWHBCII00000001 had the highest number of SNPs (67,609), whereas GWHBCII00000020 had the lowest number of SNPs (14,535). The SNPs were evenly distributed on the 20 main chromosomes, with an average of 322 SNPs per MB (data not shown).

**Table 2:** Average population genetic index values for the 50 *C. ensifolium* varieties genome simplification sequencing samples within the population.

Population	Sites	Polymorphic	HetObs	HetExp	Pi	Fis	PIC	Ne	MAF
All	1,280,516	1,280,516	0.203	0.270	0.270	0.249	0.225	1.423	0.186

The population genetic index values are averages. Population, population ID; Sites, number of SNP markers within the population; Polymorphic, number of polymorphic markers; HetObs, observed heterozygosity; HetExp, expected heterozygosity; Pi, nucleotide polymorphism within the population; Fis, average inbreeding coefficient of the population; PIC, polymorphic information content; Ne, effective number of alleles; MAF, minimum allele frequency.

### Genetic relationships and population structure analysis

**Figure 3:**
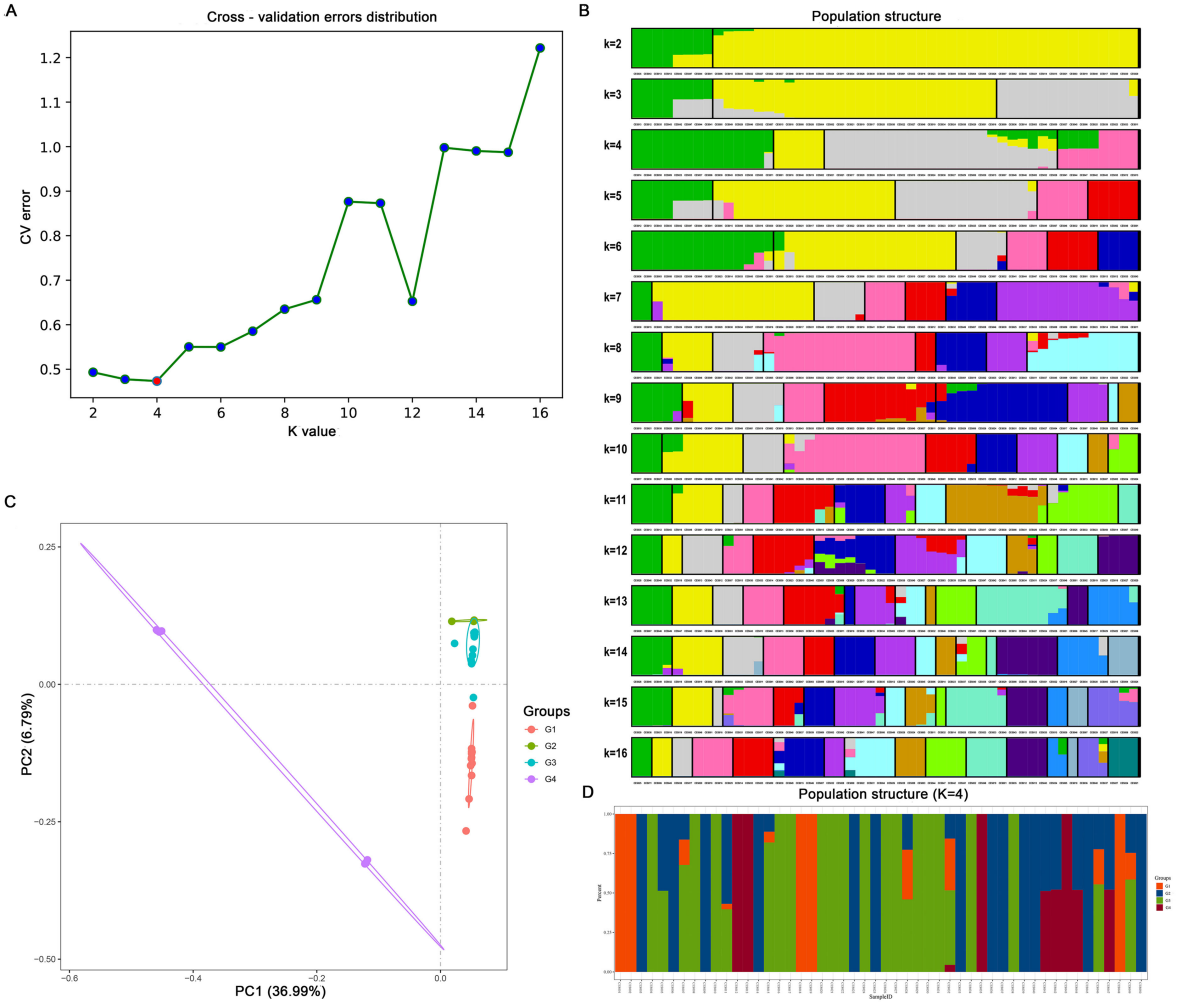
Bioinformatic analysis of 50 *C. ensifolium* varieties based on single nucleotide polymorphisms (SNPs). **(A)** The cross-validation error rate corresponding to different K values. **(B)** Population structure of 50 *C. ensifolium* varieties at different K values. The K value represents the cross-validation error rate. **(C)** A two-dimensional diagram of principal component analysis (PCA). **(D)** Population structure of 50 *C. ensifolium* samples when K = 4.

Admixture software was used to analyze the population structure of the 50 *C. ensifolium* samples. The population was divided into four subgroups (G1, G2, G3, G4) on the basis of the lowest cross-validation error rate at K=4 (**Figure 3A**). G2 included 5 varieties, which primarily descended from a common ancestor and predominantly represented purebred varieties. G4 comprised 8 varieties, predominantly hybrids, including several leaf art varieties. The G1 and G3 subgroups were more diverse, encompassing a variety of flower colors, patterns, and leaf art varieties. G1 comprised 14 varieties, with only CES011 descending from 3 ancestors, while the rest originated from a common ancestor. G3 included 23 varieties, with 16 having a single ancestral origin (**Figures 3B, D**).

PCA was conducted using GCTA software on high-quality SNPs from the 50 *C. ensifolium* samples, resulting in the samples being discriminated into four groups on the basis of the first two components, accounting for 36.99% and 6.79% of the total variation, respectively (**Figure 3C**).

**Figure 4:**
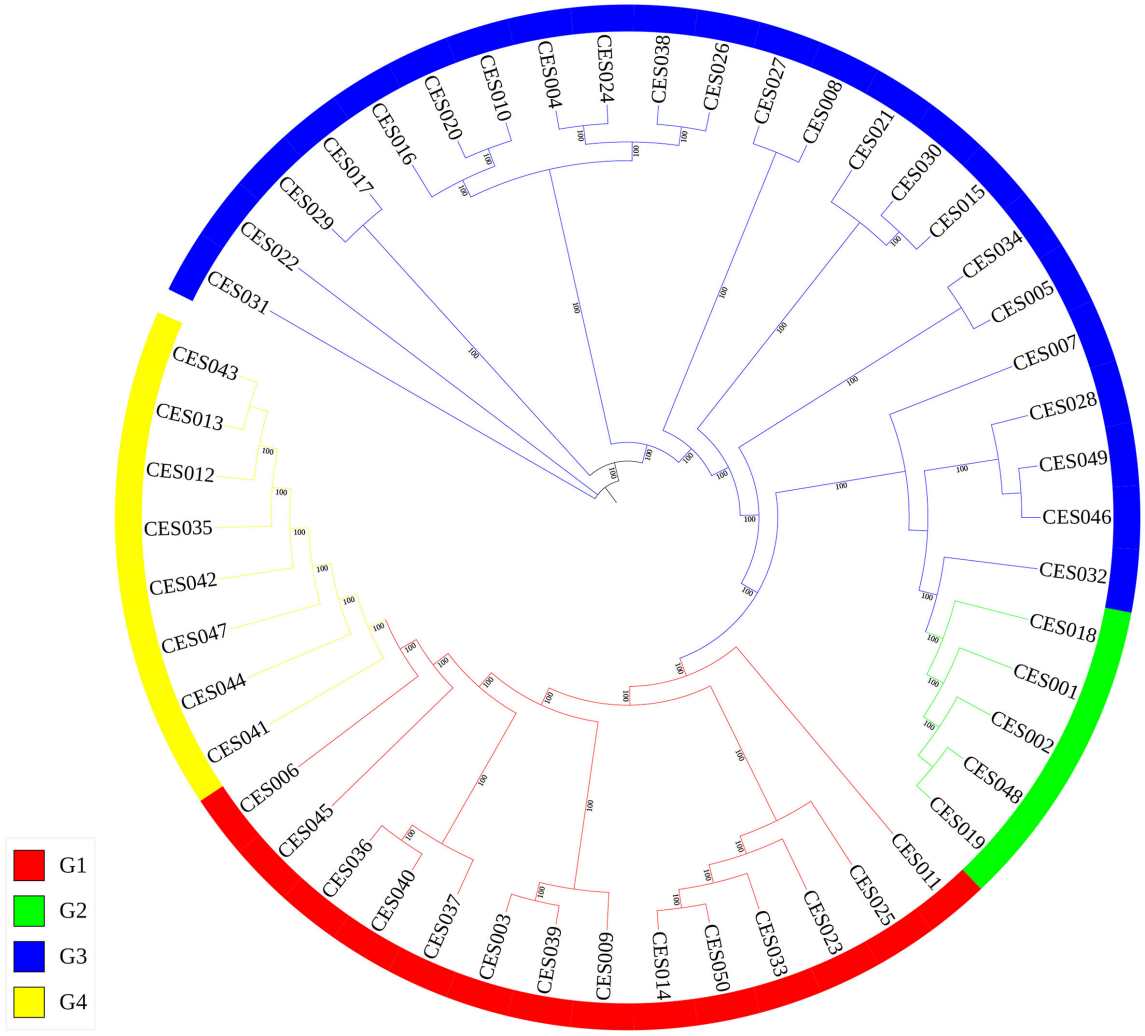
Phylogenetic tree of 50 *C. ensifolium* varieties. Branches of the same color are in the same group.

An evolutionary tree was also constructed using RAxML software, which clustered the 50 *C. ensifolium* samples into four groups (**Figure 4**), which was consistent with the PCA and population structure analysis results and was supported by high bootstrap values. The clusters presented a certain degree of similarity in terms of phenotype and origin (**Supplementary Table 1**).

### Development and verification of highly polymorphic KASP SNP markers

To develop high-quality and polymorphic KASP SNP markers that could effectively differentiate the tested samples, stringent criteria were applied, including SNP conservation and uniqueness on the chromosome DNA strands, high sequencing depth, biallelic polymorphisms, and high PIC values. A total of 7,599 SNPs were screened from 10,021,591 SNPs, with 4,360 successfully designed as KASP markers (**Supplementary Excel 2**). Among these KASP markers, 428 were located in exonic regions, accounting for 9.8% (**Supplementary Figure 2**).

**Figure 5:**
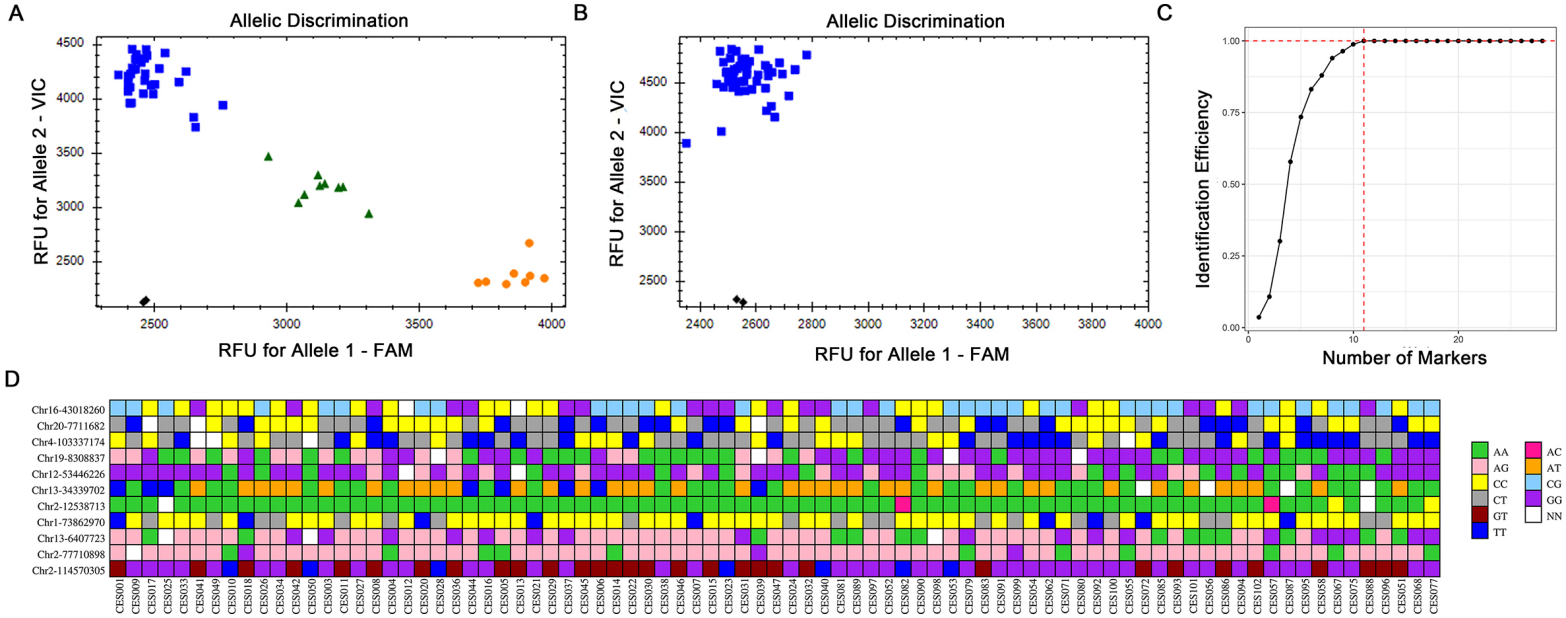
Representative KASP-labelled fluorescence assay results and fingerprint analysis of 83 *C. ensifolium* varieties. **(A)** A KASP marker with better typing. **(B)** A KASP marker with monomorphism. **(C)** Identification efficiency of the combined SNP markers. **(D)** Fingerprints of the 83 *C. ensifolium* materials. Each row represents the typing result of the same label in different samples, and each column represents a sample. The pure genotypes were AA=green, AG=light pink, CC=yellow, CT=grew, GT=dark red, TT=blue, AC=pink, AT=orange, CG=light blue, and GG=purple; and no call genotypes were designated NN=white.

To validate the practicality of the selected SNP markers, we utilized a Perl program to screen 28 loci from the 4,360 SNPs mentioned above for verification (**Supplementary Excel 4**, **Supplementary Figure 3**). The first-generation sequencing results indicated that all 28 loci were indeed present (**Table 1**). On the basis of the actual sequencing results, we subsequently optimized the primer design for KASP and conducted KASP genotyping experiments (**Supplementary Table 3**). Among the 50 simplified sequencing samples, most samples were well genotyped and effectively distinguished, but two groups (CES001, CES002, CES019, CES048; CES035, CES043) could not be differentiated (data not shown). Despite multiple rounds of screening, these two groups could not be distinguished, making the development of KASP markers difficult. Considering the cost of developing markers, this study did not continue to develop KASP primers to differentiate these similar varieties. To verify the utility of the selected KASP markers, we selected another 37 cultivated varieties of *C. ensifolium* for KASP genotyping. The results revealed that four markers (Chr12-75662664, Chr16-66004509, Chr18-11319799, Chr3-153114895) had only one genotype, whereas the other 24 markers were polymorphic and could effectively distinguish the other 37 cultivated varieties of *C. ensifolium* (**Figures 5A, B**; **Supplementary Excel 5**). These findings indicate that the selected KASP markers have good usability. Due to the large number of *C. ensifolium* varieties, by expanding the screening range these four KASP primers with consistent genotypes may still exhibit polymorphisms, thus supporting the potential value of the markers developed in this study.

### Construction of a DNA fingerprint

To protect the genetic resources of *C. ensifolium* varieties, we constructed a fingerprint map for all tested varieties on the basis of the KASP marker genotyping results, with one variety selected from each undistinguished group as a representative (CES001, CES035). A good fingerprint map requires a minimum number of markers to distinguish the maximum number of varieties to achieve simplicity, efficiency, and economy. By selecting 11 SNPs from the 28 obtained SNP loci that could distinguish 83 *C. ensifolium* varieties, we constructed a highly simplified SNP combination distributed across seven chromosomes (**Figure 5C**). Using these 11 SNPs, we successfully distinguished all 83 varieties, and there was at least one different SNP between each of the two samples. The PIC values of the 11 core KASP SNPs ranged from 0.19 to 0.37, and 90% > 0.33, indicating medium genetic diversity (**Supplementary Excel 4**). The combination of these 11 SNPs effectively distinguished the *C. ensifolium* varieties, and the fingerprint map constructed on the basis of the genotyping results further confirmed this distinction (**Figure 5D**). The remaining 17 KASP SNP markers can be used as backup markers when expanding sample testing to complement and improve the fingerprint map (**Supplementary Excel 4**).

## Discussion

### SNP-based genetic relationships among the *C. ensifolium*

*C. ensifolium* is widely distributed in southern China and is highly diverse in terms of flower color, flower type, leaf color, leaf shape, and plant morphology. As a traditional Chinese *Cymbidium* species, *C. ensifolium* is highly valued for its historical, economic, and ornamental significance (Cao et al., 2022; Ai et al., 2021). In the past, wild types with unique traits were sought from the field to breed better varieties of *C. ensifolium*. However, due to the emphasis on the protection of orchid species in China, the collection of wild genetic resources has been prohibited by law. Therefore, breeding new varieties from existing varieties has become the primary means of creating new orchid varieties. Current studies have focused mainly on the genetic control of specific traits in plants, such as flower color, leaf color, and drought resistance, providing references for future breeding techniques (Ai et al., 2023; Li et al., 2023b; Mei et al., 2024). Before new varieties are bred, it is essential to determine the genetic and kinship relationships between breeding materials (Chen et al., 2020). However, few studies have investigated the genetic and kinship relationships among commercial *C. ensifolium* varieties at the genomic level.

In this study, 50 mainstream commercial varieties of *C. ensifolium* were selected, and using ddRAD-seq technology, a series of polymorphic SNP markers were obtained through strict screening criteria. Genetic and kinship analyses of the 50 commercial varieties of *C. ensifolium* on the basis of SNP sequences were conducted. PCA and population structure analysis indicated that the 50 *C. ensifolium* varieties could be divided into 4 groups, which was also supported by the results of the maximum likelihood phylogenetic tree. The selected *C. ensifolium* varieties in this study overlap with those selected by Ai et al., but the clustering results are different, which may be due to different population constructions and segments selected for building the phylogenetic tree (Ai et al., 2019). Among the 50 selected *C. ensifolium* varieties, most originated from Taiwan, such as *C. ensifolium* var. ‘Qi Xiannv’, *C. ensifolium* var. ‘Shizhang Hong’, *C. ensifolium* var. ‘Lishan Shiwang’, *C. ensifolium* var. ‘Hong Niang’, *C. ensifolium* var. ‘Jin He’, *C. ensifolium* var. ‘Fushan Qidie’, and *C. ensifolium* var. ‘Baodao Xiannv’, which were all classified in Group 1 (G1), whereas those originating from Sichuan were mostly classified in Group 3 (G3). This finding indicates that the grouping in this study is consistent with the original sources of the varieties. Even though varieties from different regions have undergone long-term domestication, they still retain their own distinct features (Lin et al., 2022). Within the G2 group, most individuals are from the southeastern coastal regions of China. In addition to the hybrid variety *C. ensifolium* var. ‘Zhu Jin’, the other varieties also share some common phenotypic characteristics, such as flowers with light-colored sepals and labellums that are solid in color without spots. On the other hand, the G4 group consists mainly of hybrid species. Additionally, some cultivars were not grouped together with others from the same geographical origin, indicating widespread genetic exchange among cultivated varieties.

Hybrid breeding involves selecting parental varieties with a large genetic distance and distant kinship, which aids in selecting new varieties with excellent traits (Geng et al., 2021; Cui et al., 2023). The evolutionary tree in this study shows clear genetic distances and kinship relationships among the 50 C*. ensifolium* varieties. For example, *C. ensifolium* var. ‘Zhaojun Xue’, *C. ensifolium* var. ‘Da Jiangjun’, *C. ensifolium* var. ‘Huangjin Xianzi’, and *C. ensifolium* var. ‘Danxia Xiannv’ are located on the same branch and have large genetic distances from the remaining varieties, providing theoretical references for the selection of parental varieties in *C. ensifolium* breeding.

### Cultivar identification and the advantages of SNP molecular markers

*C. ensifolium* is a highly valuable ornamental and economic orchid, and through generations of breeding, there are currently an estimated thousand varieties of *C. ensifolium* available on the market (https://www.hmlan.com/auction/search-101005.htm?q=%BD%A8%C0%BC&noex=) (accessed on 5 June 2024). Identifying the correct variety of orchids to purchase online during nonflowering periods has become a challenge for many orchid enthusiasts. The traditional classification of *C. ensifolium* varieties is based mainly on morphological characteristics. Wang used 14 quantitative traits and 11 qualitative traits to describe 39 Cymbidium varieties in detail, providing a phenotypic basis for the identification of these varieties (Wang, 2020). Nevertheless, identifying varieties during nonflowering periods remains difficult. To address the shortcomings of morphological identification, researchers have also attempted to use various molecular methods for *C. ensifolium* variety identification. For example, Wang et al. successfully differentiated 9 orchid species, including *C. ensifolium*, using ALFP technology (Wang and Wang, 2014). Wang et al. also distinguished 85 *C. ensifolium* cultivars using 19 ISSR primers (Wang et al., 2011). Hu et al. differentiated 38 *C. ensifolium* varieties using 18 RAPD primers with genetic distances ranging from 0.0420 to 0.5385 [12]. Li et al. developed 55 genic-SSR polymorphic markers from the total RNA of *C. ensifolium* var. Tiegusu and distinguished 9 *Cymbidium* species and 12 *C. ensifolium* cultivars using evolutionary tree construction. The genetic distance ranged from 0.016 to 0.618 (Li et al., 2014). Although these methods can be used to differentiate *C. ensifolium* cultivars, they have limitations such as inconvenient operation, high cost, inability to distinguish genotypes, and low number of polymorphic sites, highlighting the urgent need for a new, simple, accurate, and efficient molecular identification method.

SNP molecular marker technology has numerous advantages, including a large quantity, wide distribution, allelic dimorphism, and stable inheritance (Li et al., 2023a). This technology has been successfully applied in various crops, such as rice, grape, potato, cotton, radish, and honeysuckle (Xing et al., 2024; Wang et al., 2022; Li et al., 2023a; Morales et al., 2020; Gazendam et al., 2022; Kuang et al., 2016). It is one of the marker methods recommended by the UPOV and the general guidelines for the identification of plant varieties using DNA markers (NY/T 2594-2016) (Button, 2008). However, its application in *Cymbidium* species, particularly *C. ensifolium*, is relatively limited (Yang et al., 2023).

In this study, using the ddRAD-seq technology on 50 C*. ensifolium* cultivars, a total of 10,021,591 SNP loci were obtained, surpassing the number of polymorphic markers obtained by Li et al. using genic-SSR markers in *C. ensifolium* (Li et al., 2014). On average, 964,159 SNP loci were developed per cultivar, whereas 334,967 SNP loci were obtained per *Cymbidium sinense* (Jack. ex Andr.) Willd. cultivar using SLAF-seq technology (Yang et al., 2023). These results indicate the presence of a rich SNP locus population in orchids, providing a valuable resource for developing SNP markers. After stringent filtering, 4,360 highly polymorphic SNP loci were selected, and a set of 11 SNP markers were identified that could effectively distinguish 83 C*. ensifolium* cultivars. This identification efficiency is significantly greater than that of traditional *C. ensifolium* classification methods such as AFLP, ISSR, RAPD, and genic-SSR (Wang and Wang, 2014; Wang et al., 2021a, 2011; Hu et al., 2008; Li et al., 2014).

### The main advantages of the KASP genotyping technique for identifying varieties

The Laboratory of Government Chemists (LGC) developed a high-throughput genotyping technique based mainly on SNPs based on the principle of KASP (Dipta et al., 2024). Overall, the KASP genotyping technique offers several advantages for identifying varieties. Compared with other SNP genotyping technologies, it is cost-effective, with lower material expenses per reaction (Ayalew et al., 2019; Yuan et al., 2014). Additionally, it is a simple and gel-free assay that can be easily performed using regular qPCR instruments, reducing labor costs. Compared with AFLP and SSR technology, the automatic genotyping technique, which is based on fluorescence differences, also minimizes the effect of error when the gel electrophoresis image is read (Wang and Wang, 2014; Sun et al., 2023). The design principle behind KASP primers allows quick and accurate genotyping on the basis of SNP polymorphisms, making it a valuable tool for quality control and QTL mapping (Kumar et al., 2022; Zeng et al., 2022). In conclusion, the KASP genotyping technique is a reliable and efficient method for identifying genetic variations in plant varieties.

Due to the numerous advantages of the KASP genotyping technique, KASP assays have been developed for genotyping analysis in a variety of plant species. For example, in conventional and hybrid rice, Tang et al. developed 48 KASP markers, and the 48 KASP markers had a 100% discrimination rate in 53 conventional indica varieties and 193 hybrid varieties (Tang et al., 2022). In cabbage, Li et al. selected 442 KASP SNP markers among 50 resequenced genotypes on the basis of high polymorphism information content, high minor allele frequency, wide average distribution and low heterozygosity. Using the KASP genotyping data, the genetic similarity among three kinds of inbred lines (spring cabbage, autumn cabbage and winter cabbage) was analyzed, and the heterotic groups within each ecotype were classified. Seven heterotic groups were identified for spring cabbage (77), six for autumn cabbage (70), and five for winter cabbage (97) (Li et al., 2020). In *Brassica rapa*, Hong et al. developed 100 accession-specific markers as accession-specific KASP markers. Using the results of their validation experiments, they successfully distinguished the accession-specific markers in individual accessions in test populations from noncore or commercial cultivars (Hong et al., 2022). In addition, KASP markers play important roles in genotyping and variety identification in other plants, such as apple, coffee, and wheat (Winfield et al., 2020; Patterson et al., 2017; Roncallo et al., 2019; Akpertey et al., 2020; Zhang et al., 2023b; Kumar et al., 2022). However, little research has been conducted on SNP markers and KASP marker development in *C. ensifolium*.

In our study, using ddRAD genome simplified sequencing data of 50 C*. ensifolium* commercial varieties, we obtained 10,021,591 SNP sites. After a series of strict screening criteria, we obtained 7,599 SNP sites with high polymorphism information content, high minor allele frequency, and a wide average distribution. Among these, 4,360 SNP markers were successfully converted into KASP markers, accounting for 57.4% of all 7,599 SNP markers. The conversion efficiency of KASP markers was lower than that of Cabbage (88.4%), *Capsicum annuum* L. (88.2%), and rice (94.8%) (Zhang et al., 2023b; Li et al., 2020; Yang et al., 2019). In terms of the conversion efficiency of all SNP sites into KASP sites, this study revealed an efficiency of 0.04%, which was lower than that of maize (2.42%) (Chen et al., 2021). These findings indicate that the development of KASP SNP markers for *C. ensifolium* is relatively challenging. These KASP SNP markers provide a rich resource database of polymorphic sites for the identification and consistency testing of *C. ensifolium* varieties and even orchid varieties.

### Identification and validation of 83 *C. ensifolium* germplasm resources by SNP fingerprints

Germplasm resources, as important biological resources, constitute the genetic basis for breeding high-quality and unique new varieties (Wang et al., 2021b; Li et al., 2023a). With the development of technology, the types and quantity of discovered germplasm resources are becoming increasingly diverse and abundant. Conducting DNA fingerprinting on germplasm resources is a good choice for effective protection and efficient utilization of many germplasm resources. DNA fingerprinting was first proposed by the geneticist Alec Jeffreys from the University of Leicester in 1985. DNA fingerprinting uses isolated human microsatellite DNA as a gene probe, hybridizes it with enzyme-cut fragments of human nuclear DNA, and obtains hybrid bands composed of alleles from multiple loci of different lengths. These fingerprints are unique to each individual, similar to human fingerprints (Jeffreys, 2013). The construction of a DNA fingerprint map can provide each germplasm with a unique identity, assist in accurate identification of germplasm resources, and play an important role in variety specificity and authenticity, seed purity identification, improved resource utilization efficiency, and protection of the intellectual property of plant breeding (Xing et al., 2024; Shen et al., 2021; Yang et al., 2022; Tian et al., 2021). SNP molecular markers are widely used for constructing DNA fingerprint maps in various plants, such as maize, cucumber, honeysuckle, cigar tobacco, radish, and red raspberry (Mannino et al., 2023; Zhang et al., 2024; Wei et al., 2024; Li et al., 2023a; Wang et al., 2021b; Tian et al., 2021; Xing et al., 2024; Clare et al., 2023; Zhang et al., 2022a). The accuracy of SNP fingerprinting has been validated in cucumber through distinctness, uniformity, and stability (DUS) testing, further demonstrating the accuracy and practicality of SNP fingerprinting (Zhang et al., 2022a). However, there have been no reports of SNP fingerprints of *C. ensifolium* germplasm resources.

SNP molecular markers are widely used for gene identification, germplasm characterization, and variety fingerprinting (Yang and Zhu, 2015; Chen et al., 2022; Magbanua et al., 2023; Zhao et al., 2017). However, it has been reported that only a small proportion of SNP loci can be selected and genotyped successfully. The authenticity of SNP sites in simplified floral genome sequencing is low, making the validation of SNP site authenticity even more important for constructing a fingerprint map (Liu et al., 2022a). Our study also revealed a low authenticity of SNP sites in simplified sequencing sites, leading to difficulties in KASP primer development. Using 50 simplified sequencing varieties, KASP primers were developed using Perl programs, resulting in 28 KASP markers. Most *C. ensifolium* varieties could be easily distinguished, but there were still two groups that could not be differentiated even after multiple KASP primer developments, namely, CES001, CES002, CES019, and CES048 and CES035, and CES043. This may be due to the limited number of 28 KASP-SNP markers and the limited genomic variations that can be revealed, necessitating the development of more *C. ensifolium* KASP-SNP markers. Additionally, the simplified genome data used for marker development cover only approximately 2% of the genome, limiting the variation that can be revealed. Furthermore, the two groups may be closely related, with minimal genetic differences (Mei, 2023). Therefore, one representative variety (CES001 and CES035) was selected from each group for fingerprint construction. Furthermore, 37 commercially cultivated *C. ensifolium* varieties were added to validate the utility of the KASP markers. These 28 markers were successfully used for genotyping 83 commercial *C. ensifolium* varieties.

The construction of a molecular fingerprint map of germplasm resources requires the use of a minimal number of primers to differentiate the maximum number of germplasms. Therefore, selecting appropriate primers is an important prerequisite for constructing a molecular fingerprint map (Wang et al., 2021b; Li et al., 2023a). Based on the principle of using the minimum number of markers to differentiate the maximum number of varieties, 11 KASP markers were selected from 28 KASP markers (PIC: 0.195-0.375; MAF: 0.125-0.479; HE: 0.219-0.499), which could efficiently differentiate all *83 C. ensifolium* commercial varieties with at least one genotypic difference. On average, one SNP locus could identify 8 varieties, which is higher than the SNP marker differentiation efficiency developed in Chinese flowering cabbage (18 core SNP markers could completely differentiate all 89 cabbage varieties) and melon (40 core SNP markers efficiently differentiate 99% of the 259 commercial melon varieties) (Ren et al., 2023; Zhang et al., 2023a). However, it is lower than the SNP marker differentiation efficiency developed in radish, which has reached 24 accessions per SNP locus (15 core SNP markers could completely differentiate all 356 radish varieties) (Xing et al., 2024). This may be attributed to the relatively high quantity and quality of the genomic database used for radish SNP marker development (Xing et al., 2024). The development of radish markers has demonstrated the great potential and advantages of using SNP loci to differentiate varieties, prompting us to continuously expand the genomic database of *C. ensifolium* to develop more efficient SNP markers.

It is noted that there are some varieties that cannot be distinguished when using SNP loci to differentiate species, which has been observed in crops like melon and cigar tobacco (Zhang et al., 2023a; Wang et al., 2021b). In a study on cigar tobacco, Yanyan et al. utilized 47 core KASP markers to differentiate 216 cigar tobacco germplasm resources, and found that some varieties could not be distinguished. Through phenotype analysis, these were identified as synonyms (Wang et al., 2021b). The phenomenon of synonyms also exists in the *C. ensifolium* market, where vendors may alter the phenotype of orchids through physical or chemical treatments to create fake new varieties, and rename existing varieties arbitrarily (Ning, 2022; Li, 1994). This confusion in commercial varieties is detrimental to the conservation and breeding of orchids, and SNP marker-based variety identification can greatly regulate the *C. ensifolium* variety market. In cases where differentiation is not possible, DUS determination (NY/T 2441-2013 Guidelines for the conduct of tests for distinctness, uniformity and stability *Cymbidium*) can be used for further confirmation of varieties.

The PIC value is considered the most important indicator of the usefulness of molecular markers. Markers with high PIC values are usually highly polymorphic, whereas markers with low PIC values are considered less polymorphic. Furthermore, markers with PIC values >0.5 are usually considered highly polymorphic, those with PIC values of 0.25<PIC ≤ 0.5 are usually considered medium polymorphic, and those with PIC values ≤0.25 are usually considered lowly polymorphic (Liu et al., 2022b). The 11 core markers required for fingerprint map construction had a PIC range of 0.195-0.375, with 90% > 0.336 indicating medium polymorphism, demonstrating the practicality and reliability of fingerprint map construction. Therefore, the 11 KASP markers are clearly reliable, effective, and accurate in detecting 83 *C. ensifolium* germplasm resources.

However, these 83 *C. ensifolium* commercial varieties constitute only a small portion of the total commercial varieties of *C. ensifolium*. To distinguish total commercial varieties, especially similar varieties, more SNP markers with greater discriminatory ability are necessary. Although only 28 out of 4,360 KASP markers were verified, verifying the remaining markers could identify more high-quality markers. These 83 commercial varieties include various typical shapes of *C. ensifolium* commercial varieties, as well as newly bred hybrid varieties in China in recent years. Additionally, utilizing SNP molecular markers and fingerprints comprehensively can effectively improve the identification capabilities of *C. ensifolium* varieties containing wild varieties, which is essential for wild resource protection, and further research is needed in this area.

## Conclusion

This study provides comprehensive information about the genetic diversity of *C. ensifolium* commercial cultivars in China on the basis of a population of 50 *C. ensifolium* commercial cultivars. A series of SNPs were discovered by ddRAD-seq of 50 diverse *C. ensifolium* commercial cultivars, and the SNPs were converted into KASP panels for the genotyping of a large set of *C. ensifolium* commercial cultivars. Phylogenetic and PCA analyses revealed that the 50 *C. ensifolium* commercial cultivars were divided into four well-separated clusters, and a correlation was observed between the group distribution and the geographical origin of the *C. ensifolium* germplasm. A set of 28 KASP SNP markers was screened, and a minimum set of 11 KASP SNP markers (with 90% PIC values>0.336) was verified to distinguish 83 *C. ensifolium* commercial cultivars completely. The 11 SNP genotypes of each *C. ensifolium* variety were used to generate SNP fingerprints of a major collection (83) of cultivated *C. ensifolium* varieties in China. The KASP markers developed in this study could also be utilized for evaluating the variety authenticity of *C. ensifolium* cultivars. This is the first study to measure the diversity and population structure of a large collection of *C. ensifolium* in China on the basis of SNPs from simplified genome sequencing and the first application of KASP techniques in *C. ensifolium* for genetic studies. This is also the first study to construct a fingerprint chart of *C. ensifolium* commercial cultivars using SNP markers. The information generated in this study will aid in the selection of suitable genotypes for the breeding of new cultivars and provide a scientific basis and technical support for the protection and identification of new *C. ensifolium* cultivars and wild germplasm resources.

## Data Availability

The datasets generated for this study can be found in the NCBI database (BioProject accession number: PRJNA1127271). The original contributions presented in the study are included in the article/**Supplementary Material**. Further inquiries can be directed to the corresponding author.

## Supplementary material

Supplementary Excel 1 Information on 28 *C. ensifolium* KASP marker primers.

Supplementary Excel 2 Information on 4360 *C. ensifolium* SNP loci.

Supplementary Excel 3 Statistical table of the number of variations in the SNP type.

Supplementary Excel 4 Genetic diversity information of 28 KASP markers in *C. ensifolium* materials.

Supplementary Excel 5 KASP genotyping results of 28 markers for 83 *C. ensifolium* varieties.
