# Supplementary material for: Development of KASP markers, SNP fingerprinting and population genetic analysis of Cymbidium ensifolium (L.) Sw. germplasm resources in China
Source: Front Plant Sci. 2025 Jan 8;15:1460603. doi: 10.3389/fpls.2024.1460603 (PMC11750851; doi:10.3389/fpls.2024.1460603)
Supplement: Supplementary file 1 [file DataSheet1.docx]

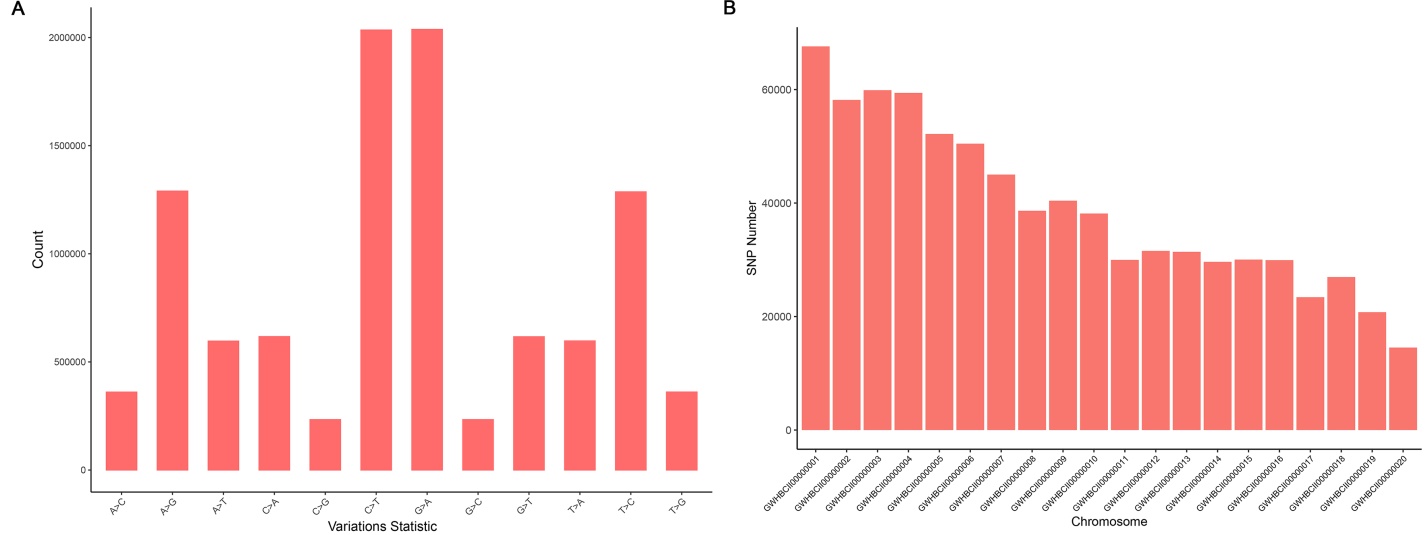


Supplementary Figure 1

Single nucleotide polymorphism (SNP) identiﬁcation of 50 *C. ensifolium* samples. (A) Number of SNP types. The horizontal axis represents the different types of SNP mutations, and the vertical axis represents the number of mutations. (B) Number of SNPs on each chromosome. The horizontal axis represents the chromosome number, and the vertical axis represents the number of SNPs.


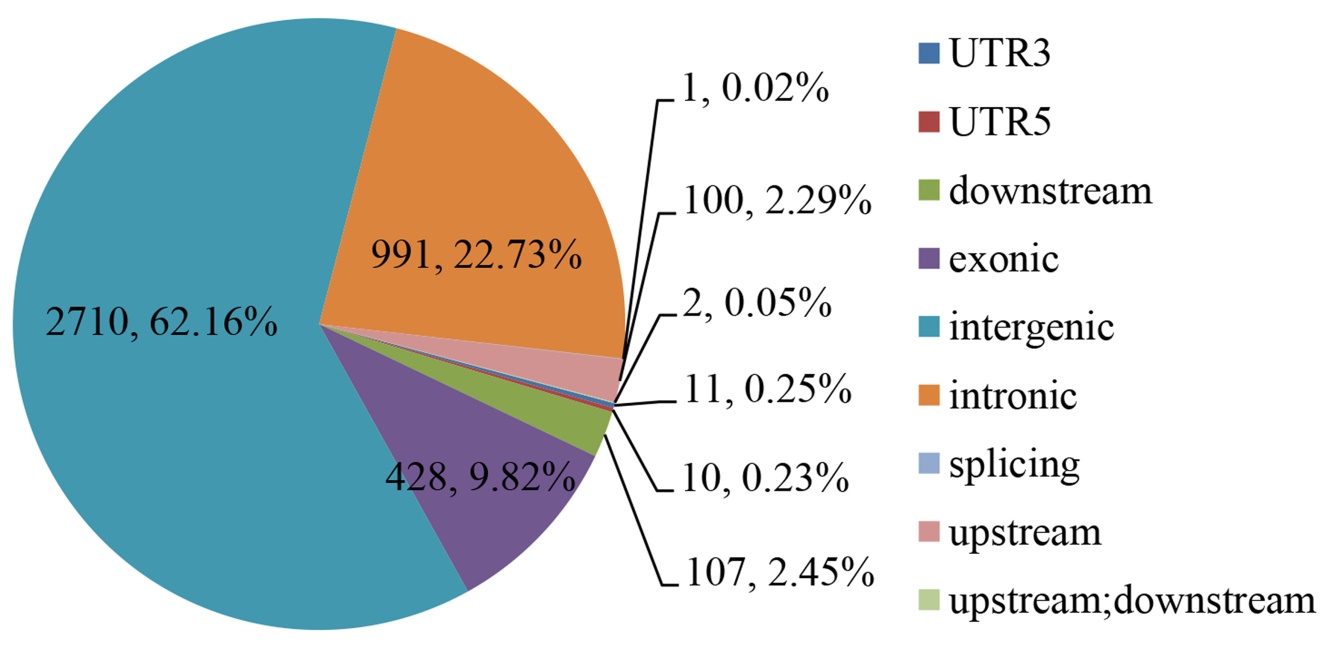


Supplementary Figure 2

Label distribution map of successful KASP primer design


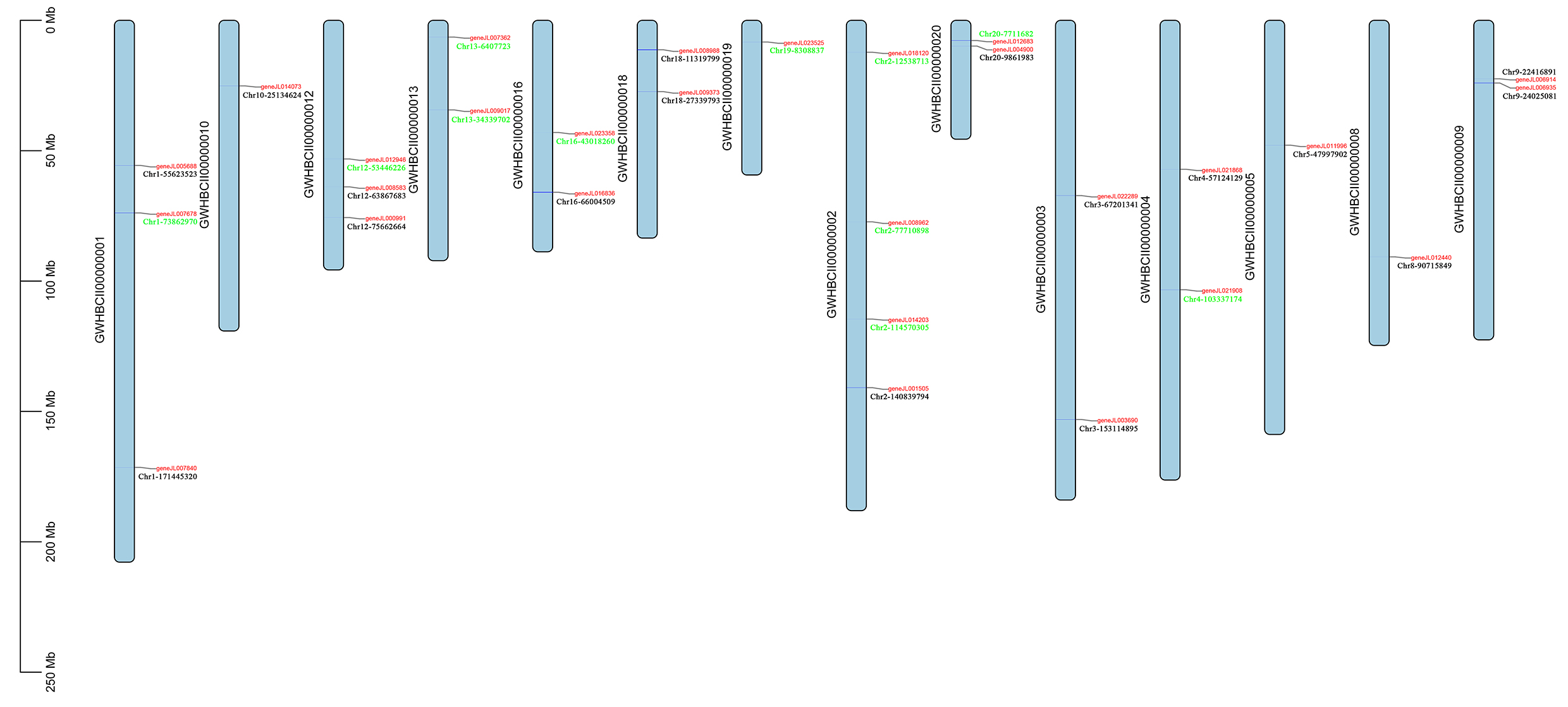


Supplementary Figure 3

The distribution of 28 KASP markers among *C. ensifolium* chromosomes. The 11 core KASP markers selected for fingerprinting of *C. ensifolium* cultivars highlighted in green. The red markers indicate the genes where the KASP markers are located.
