# Supplementary material for: Development of KASP markers, SNP fingerprinting and population genetic analysis of Cymbidium ensifolium (L.) Sw. germplasm resources in China
Source: Front Plant Sci. 2025 Jan 8;15:1460603. doi: 10.3389/fpls.2024.1460603 (PMC11750851; doi:10.3389/fpls.2024.1460603)
Supplement: Supplementary file 2 [file DataSheet2.docx]

**Supplementary Table 1** Fifty *C. ensifolium* varieties used for genome simplification sequencing and preliminary screening of KASP markers.

| **Serial number** | **Variety number** | **Grouping** | **Place of origin** | **Variety Name** | [**Source**](javascript:;) |
| --- | --- | --- | --- | --- | --- |
| 1 | CES003 | G1 | Guangdong, China | *C. ensifolium* var. ‘Gaoshan Chunse’ | C |
| 2 | CES006 | G1 | Taiwan, China | *C. ensifolium* var. ‘Qi Xiannv’ | A |
| 3 | CES009 | G1 | Fujian, China | *C. ensifolium* var. ‘Tao Jiao’ | A |
| 4 | CES011 | G1 | Taiwan, China | *C. ensifolium* var. ‘Shizhang Hong’ | A |
| 5 | CES014 | G1 | Fujian, China | *C. ensifolium* var. ‘Zhaoyang Sanxing’ | A |
| 6 | CES023 | G1 | Taiwan, China | *C. ensifolium* var. ‘Lishan Shiwang’ | B |
| 7 | CES025 | G1 | Hybrid variety, China | *C. ensifolium* var. ‘Hongmao Ruidie’ | B |
| 8 | CES033 | G1 | Taiwan, China | *C. ensifolium* var. ‘Hong Niang’ | B |
| 9 | CES036 | G1 | Taiwan, China | *C. ensifolium* var. ‘Jin He’ | A |
| 10 | CES037 | G1 | Taiwan, China | *C. ensifolium* var. ‘Fushan Qidie’ | A |
| 11 | CES039 | G1 | Sichuan, China | *C. ensifolium* var. ‘Siji Emei’ | A |
| 12 | CES040 | G1 | Fujian, China | *C. ensifolium* var. ‘Qicai Linglong’ | A |
| 13 | CES045 | G1 | Taiwan, China | *C. ensifolium* var. ‘Baodao Xiannv’ | A |
| 14 | CES050 | G1 | Fujian, China | *C. ensifolium* var. ‘Huangjin Caihong’ | A |
| 15 | CES001 | G2 | Hong Kong, China | *C. ensifolium* var. ‘Qingshan Yuquan’ | B |
| 16 | CES002 | G2 | Guangdong, China | *C. ensifolium* var. ‘Zhude Su’ | C |
| 17 | CES018 | G2 | Hybrid variety, China | *C. ensifolium* var. ‘Zhu Jin’ | B |
| 18 | CES019 | G2 | Fujian, China | *C. ensifolium* var. ‘Wolong Su’ | B |
| 19 | CES048 | G2 | Fujian, China | *C. ensifolium* var. ‘Tianxiang Su’ | A |
| 20 | CES004 | G3 | Sichuan, China | *C. ensifolium* var. ‘He Wang’ | C |
| 21 | CES005 | G3 | Hybrid variety, China | *C. ensifolium* var. ‘Dongfang Honghe’ | A |
| 22 | CES007 | G3 | Taiwan, China | *C. ensifolium* var. ‘Yihong Shuangjiao’ | A |
| 23 | CES008 | G3 | Sichuan, China | *C. ensifolium* var. ‘Yi Pinmei’ | A |
| 24 | CES010 | G3 | Sichuan, China | *C. ensifolium* var. ‘Cuiyu Mudan’ | A |
| 25 | CES015 | G3 | Sichuan, China | *C. ensifolium* var. ‘Huangguangdeng Mei’ | B |
| 26 | CES016 | G3 | Sichuan, China | *C. ensifolium* var. ‘Huang Yipin’ | B |
| 27 | CES017 | G3 | Sichuan, China | *C. ensifolium* var. ‘Hong Yipin’ | B |
| 28 | CES020 | G3 | Sichuan, China | *C. ensifolium* var. ‘Mo Bao’ | B |
| 29 | CES021 | G3 | Sichuan, China | *C. ensifolium* var. ‘Wuue Qilin’ | B |
| 30 | CES022 | G3 | Sichuan, China | *C. ensifolium* var. ‘Caiyun Zhuiyue’ | B |
| 31 | CES024 | G3 | Sichuan, China | *C. ensifolium* var. ‘Chuichui Die’ | B |
| 32 | CES026 | G3 | Sichuan, China | *C. ensifolium* var. ‘Zhonghua Shuixian’ | B |
| 33 | CES027 | G3 | Chengdu, China | *C. ensifolium* var. ‘Jingcai Mei’ | B |
| 34 | CES028 | G3 | Guangdong, China | *C. ensifolium* var. ‘Liuyang He’ | B |
| 35 | CES029 | G3 | Sichuan, China | *C. ensifolium* var. ‘Xia Huangmei’ | B |
| 36 | CES030 | G3 | Sichuan, China | *C. ensifolium* var. ‘Lvguangdeng Mei’ | B |
| 37 | CES031 | G3 | Sichuan, China | *C. ensifolium* var. ‘Emei Xian’ | B |
| 38 | CES032 | G3 | Fujian, China | *C. ensifolium* var. ‘Jin Qi’ | A |
| 39 | CES034 | G3 | Sichuan, China | *C. ensifolium* var. ‘Jun He’ | A |
| 40 | CES038 | G3 | Sichuan, China | *C. ensifolium* var. ‘Juguo Huanqing’ | B |
| 41 | CES046 | G3 | Fujian, China | *C. ensifolium* var. ‘Bai Niangzi’ | A |

**Supplementary Table 1 (continued)**

| **Serial number** | **Variety number** | **Grouping** | **Place of origin** | **Variety Name** | **Source** |
| --- | --- | --- | --- | --- | --- |
| 42 | CES049 | G3 | Fujian, China | *C. ensifolium* var. ‘Jinsi Mawei’ | A |
| 43 | CES012 | G4 | Hybrid variety, China | *C. ensifolium* var. ‘Da Jiangjun’ | A |
| 44 | CES013 | G4 | Hybrid variety, China | *C. ensifolium* var. ‘Huangjin Xianzi’ | A |
| 45 | CES035 | G4 | Hybrid variety, China | *C. ensifolium* var. ‘Zhaojun Xue’ | A |
| 46 | CES041 | G4 | Hybrid variety, China | *C. ensifolium* var. ‘Jinjian Qidie’ | A |
| 47 | CES042 | G4 | Hybrid variety,China | *C. ensifolium* var. ‘Hong Pao’ | A |
| 48 | CES043 | G4 | Guangdong, China | *C. ensifolium* var. ‘Danxia Xiannv’ | A |
| 49 | CES044 | G4 | Hybrid variety, China | *C. ensifolium* var. ‘Da Guoguan’ | A |
| 50 | CES047 | G4 | Hybrid variety, China | *C. ensifolium* var. ‘Yuye Yaoji’ | A |

Note: *C. ensifolium* varieties were purchased from the following companies: A: Zhangzhou Gaozhai Agricultural Development Co., Ltd.; B: Zhangzhou city Nanjing County Shili Lanxiang orchid garden; C: Liuyang Huiming orchid planting professional cooperative.

**Supplementary Table 2** Eighty-three *C. ensifolium* varieties used for constructing fingerprint maps and rescreening KASP markers.

| **Serial number** | **Variety number** | **Variety Name** | [**Source**](javascript:;) |
| --- | --- | --- | --- |
| 1 | CES001 | *C. ensifolium* var. ‘Qingshan Yuquan’ | B |
| 2 | CES003 | *C. ensifolium* var. ‘Gaoshan Chunse’ | C |
| 3 | CES004 | *C. ensifolium* var. ‘He Wang’ | C |
| 4 | CES005 | *C. ensifolium* var. ‘Dongfang Honghe’ | A |
| 5 | CES006 | *C. ensifolium* var. ‘Qi Xiannv’ | A |
| 6 | CES007 | *C. ensifolium* var. ‘Yihong Shuangjiao’ | A |
| 7 | CES008 | *C. ensifolium* var. ‘Yi Pinmei’ | A |
| 8 | CES009 | *C. ensifolium* var. ‘Tao Jiao’ | A |
| 9 | CES010 | *C. ensifolium* var. ‘Cuiyu Mudan’ | A |
| 10 | CES011 | *C. ensifolium* var. ‘Shizhang Hong’ | A |
| 11 | CES012 | *C. ensifolium* var. ‘Da Jiangjun’ | A |
| 12 | CES013 | *C. ensifolium* var. ‘Huangjin Xianzi’ | A |
| 13 | CES014 | *C. ensifolium* var. ‘Zhaoyang Sanxing’ | A |
| 14 | CES015 | *C. ensifolium* var. ‘Huangguangdeng Mei’ | B |
| 15 | CES016 | *C. ensifolium* var. ‘Huang Yipin’ | B |
| 16 | CES017 | *C. ensifolium* var. ‘Hong Yipin’ | B |
| 17 | CES018 | *C. ensifolium* var. ‘Zhu Jin’ | B |
| 18 | CES020 | *C. ensifolium* var. ‘Mo Bao’ | B |
| 19 | CES021 | *C. ensifolium* var. ‘Wuue Qilin’ | B |
| 20 | CES022 | *C. ensifolium* var. ‘Caiyun Zhuiyue’ | B |
| 21 | CES023 | *C. ensifolium* var. ‘Lishan Shiwang’ | B |
| 22 | CES024 | *C. ensifolium* var. ‘Chuichui Die’ | B |
| 23 | CES025 | *C. ensifolium* var. ‘Hongmao Ruidie’ | B |
| 24 | CES026 | *C. ensifolium* var. ‘Zhonghua Shuixian’ | B |
| 25 | CES027 | *C. ensifolium* var. ‘Jingcai Mei’ | B |
| 26 | CES028 | *C. ensifolium* var. ‘Liuyang He’ | B |
| 27 | CES029 | *C. ensifolium* var. ‘Xia Huangmei’ | B |
| 28 | CES030 | *C. ensifolium* var. ‘Lvguangdeng Mei’ | B |
| 29 | CES031 | *C. ensifolium* var. ‘Emei Xian’ | B |
| 30 | CES032 | *C. ensifolium* var. ‘Jin Qi’ | A |
| 31 | CES033 | *C. ensifolium* var. ‘Hong Niang’ | B |
| 32 | CES034 | *C. ensifolium* var. ‘Jun He’ | A |
| 33 | CES035 | *C. ensifolium* var. ‘Zhaojun Xue’ | A |
| 34 | CES036 | *C. ensifolium* var. ‘Jin He’ | A |
| 35 | CES037 | *C. ensifolium* var. ‘Fushan Qidie’ | A |
| 36 | CES038 | *C. ensifolium* var. ‘Juguo Huanqing’ | B |
| 37 | CES039 | *C. ensifolium* var. ‘Siji Emei’ | A |
| 38 | CES040 | *C. ensifolium* var. ‘Qicai Linglong’ | A |
| 39 | CES041 | *C. ensifolium* var. ‘Jinjian Qidie’ | A |
| 40 | CES042 | *C. ensifolium* var. ‘Hong Pao’ | A |
| 41 | CES044 | *C. ensifolium* var. ‘Da Guoguan’ | A |

**Supplementary Table 2 (continued)**

| **Serial number** | **Variety number** | **Variety Name** | [**Source**](javascript:;) |
| --- | --- | --- | --- |
| 42 | CES045 | *C. ensifolium* var. ‘Baodao Xiannv’ | A |
| 43 | CES046 | *C. ensifolium* var. ‘Bai Niangzi’ | A |
| 44 | CES047 | *C. ensifolium* var. ‘Yuye Yaoji’ | A |
| 45 | CES049 | *C. ensifolium* var. ‘Jinsi Mawei’ | A |
| 46 | CES050 | *C. ensifolium* var. ‘Huangjin Caihong’ | A |
| 47 | CES051 | *C. ensifolium* var. ‘Ban Jianghong’ | B |
| 48 | CES052 | *C. ensifolium* var. ‘Mushan Zi’ | B |
| 49 | CES053 | *C. ensifolium* var. ‘Xiao Fengxian’ | B |
| 50 | CES054 | *C. ensifolium* var. ‘Riyuetan Zhilian’ | B |
| 51 | CES056 | *C. ensifolium* var. ‘Yan Zhihong’ | B |
| 52 | CES057 | *C. ensifolium* var. ‘Hong Baoshi’ | B |
| 53 | CES058 | *C. ensifolium* var. ‘Zixia Xianzi’ | B |
| 54 | CES062 | *C. ensifolium* var. ‘Chanyue Damo’ | A |
| 55 | CES067 | *C. ensifolium* var. ‘Taibei Xiaojie’ | A |
| 56 | CES068 | *C. ensifolium* var. ‘Hong Shuangxi’ | A |
| 57 | CES071 | *C. ensifolium* var. ‘Hong Jiangjun’ | A |
| 58 | CES072 | *C. ensifolium* var. ‘Xiangxiang Suxin’ | A |
| 59 | CES075 | *C. ensifolium* var. ‘Huangjin Zhua’ | A |
| 60 | CES077 | *C. ensifolium* var. ‘Xiao Meiren’ | A |
| 61 | CES079 | *C. ensifolium* var. ‘Huangjin Meiren’ | A |
| 62 | CES080 | *C. ensifolium* var. ‘Babao Qizhen’ | B |
| 63 | CES081 | *C. ensifolium* var. ‘Yibin Hexian’ | B |
| 64 | CES082 | *C. ensifolium* var. ‘Xianshan Hong’ | B |
| 65 | CES083 | *C. ensifolium* var. ‘Ruyi Honghe’ | B |
| 66 | CES085 | *C. ensifolium* var. ‘Fu Long’ | B |
| 67 | CES086 | *C. ensifolium* var. ‘Minshan Yanzhi’ | B |
| 68 | CES087 | *C. ensifolium* var. ‘Datang Gongfen’ | B |
| 69 | CES088 | *C. ensifolium* var. ‘Hao Rizi’ | B |
| 70 | CES089 | *C. ensifolium* var. ‘Hong Sanniang’ | B |
| 71 | CES090 | *C. ensifolium* var. ‘Man Tanghong’ | B |
| 72 | CES091 | *C. ensifolium* var. ‘Hongchan Mudan’ | B |
| 73 | CES092 | *C. ensifolium* var. ‘Huicui Fenhe’ | B |
| 74 | CES093 | *C. ensifolium* var. ‘Huang Di’ | B |
| 75 | CES094 | *C. ensifolium* var. ‘Lijiang Xue’ | B |
| 76 | CES095 | *C. ensifolium* var. ‘Dangong Yuhe’ | B |
| 77 | CES096 | *C. ensifolium* var. ‘Hua Xianzi’ | B |
| 78 | CES097 | *C. ensifolium* var. ‘Qiu Xuesu’ | B |
| 79 | CES098 | *C. ensifolium* var. ‘Foguang Hongsu’ | B |
| 80 | CES099 | *C. ensifolium* var. ‘Qingshen Mei’ | B |
| 81 | CES100 | *C. ensifolium* var. ‘Junlin Tianxia’ | B |
| 82 | CES101 | *C. ensifolium* var. ‘Su Junhe’ | B |
| 83 | CES102 | *C. ensifolium* var. ‘Tiegu Su’ | D |

Note: C. ensifolium varieties were purchased from the following companies: A: Zhangzhou Gaozhai Agricultural Development Co., Ltd.; B: Zhangzhou city Nanjing County Shili Lanxiang orchid garden; C: Liuyang Huiming orchid planting professional cooperative; D: Hunan Lanyuan Agriculture and Forestry Technology Development Co., Ltd.

**Supplementary Table 3** Statistical evaluation of sequencing data for simplified *C. ensifolium* genome sequencing samples.

| **Variety number** | **Read Number** | **Base Number（bp）** | **A(%)** | **T(%)** | **G(%)** | **C(%)** | **GC**  **(%)** | **Q30**  **(%)** | **Q20**  **(%)** | **Average**  **Q** |
| --- | --- | --- | --- | --- | --- | --- | --- | --- | --- | --- |
| CES001 | 4,420,842 | 1,273,202,496 | 32.71 | 32.84 | 16.96 | 17.49 | 34.45 | 92.42 | 97.56 | 35.75 |
| CES002 | 4,777,737 | 1,375,988,256 | 32.87 | 32.97 | 16.82 | 17.34 | 34.16 | 94.16 | 98.21 | 36.05 |
| CES003 | 3,726,946 | 1,073,360,448 | 32.38 | 32.81 | 17.19 | 17.62 | 34.81 | 94.00 | 98.11 | 36.01 |
| CES004 | 4,997,952 | 1,439,410,176 | 32.73 | 32.92 | 16.94 | 17.41 | 34.35 | 94.15 | 98.18 | 36.04 |
| CES005 | 5,420,063 | 1,560,978,144 | 32.76 | 32.84 | 16.92 | 17.47 | 34.39 | 93.96 | 98.13 | 36.01 |
| CES006 | 4,254,007 | 1,225,154,016 | 32.75 | 32.93 | 16.90 | 17.42 | 34.32 | 94.07 | 98.16 | 36.03 |
| CES007 | 4,813,924 | 1,386,410,112 | 32.54 | 32.78 | 17.07 | 17.61 | 34.68 | 94.03 | 98.14 | 36.02 |
| CES008 | 5,024,406 | 1,447,028,928 | 32.72 | 32.86 | 16.94 | 17.48 | 34.42 | 94.16 | 98.20 | 36.05 |
| CES009 | 4,771,462 | 1,374,181,056 | 32.74 | 32.83 | 16.93 | 17.49 | 34.42 | 94.19 | 98.22 | 36.05 |
| CES010 | 4,660,335 | 1,342,176,480 | 32.51 | 32.73 | 17.15 | 17.61 | 34.76 | 94.03 | 98.14 | 36.02 |
| CES011 | 4,353,767 | 1,253,884,896 | 32.43 | 32.61 | 17.23 | 17.72 | 34.96 | 93.69 | 98.01 | 35.96 |
| CES012 | 4,717,759 | 1,358,714,592 | 32.65 | 32.87 | 16.97 | 17.51 | 34.48 | 94.05 | 98.14 | 36.03 |
| CES013 | 4,710,495 | 1,356,622,560 | 32.63 | 32.81 | 17.01 | 17.55 | 34.56 | 94.11 | 98.17 | 36.04 |
| CES014 | 3,745,721 | 1,078,767,648 | 32.44 | 32.62 | 17.22 | 17.72 | 34.94 | 94.10 | 98.17 | 36.04 |
| CES015 | 4,596,949 | 1,323,921,312 | 32.39 | 32.59 | 17.27 | 17.75 | 35.02 | 93.91 | 98.09 | 36.00 |
| CES016 | 3,701,355 | 1,065,990,240 | 32.30 | 32.53 | 17.34 | 17.83 | 35.17 | 94.08 | 98.17 | 36.03 |
| CES017 | 3,614,872 | 1,041,083,136 | 32.59 | 32.85 | 17.04 | 17.52 | 34.56 | 93.99 | 98.13 | 36.02 |
| CES018 | 4,078,873 | 1,174,715,424 | 32.74 | 32.77 | 16.98 | 17.50 | 34.49 | 93.75 | 98.07 | 35.98 |
| CES019 | 5,161,771 | 1,486,590,048 | 32.67 | 32.83 | 16.98 | 17.51 | 34.50 | 94.08 | 98.16 | 36.03 |
| CES020 | 4,127,490 | 1,188,717,120 | 32.46 | 32.60 | 17.21 | 17.72 | 34.93 | 94.26 | 98.25 | 36.07 |
| CES021 | 4,913,616 | 1,415,121,408 | 32.75 | 32.74 | 17.00 | 17.51 | 34.51 | 90.56 | 96.60 | 35.39 |
| CES022 | 5,200,919 | 1,497,864,672 | 32.73 | 32.80 | 16.97 | 17.50 | 34.47 | 92.41 | 97.39 | 35.72 |
| CES023 | 5,813,889 | 1,674,400,032 | 32.77 | 32.85 | 16.92 | 17.45 | 34.37 | 92.42 | 97.40 | 35.73 |
| CES024 | 4,185,183 | 1,205,332,704 | 32.61 | 32.76 | 17.07 | 17.55 | 34.62 | 92.54 | 97.44 | 35.75 |
| CES025 | 4,433,220 | 1,276,767,360 | 32.69 | 32.79 | 17.01 | 17.50 | 34.51 | 92.25 | 97.33 | 35.70 |
| CES026 | 5,285,916 | 1,522,343,808 | 32.72 | 32.80 | 16.98 | 17.50 | 34.48 | 92.41 | 97.39 | 35.72 |
| CES027 | 5,212,183 | 1,501,108,704 | 32.78 | 32.80 | 16.95 | 17.47 | 34.43 | 92.31 | 97.35 | 35.71 |
| CES028 | 4,745,471 | 1,366,695,648 | 32.65 | 32.74 | 17.07 | 17.54 | 34.61 | 92.42 | 97.38 | 35.72 |
| CES029 | 4,807,239 | 1,384,484,832 | 32.74 | 32.85 | 16.95 | 17.46 | 34.41 | 92.46 | 97.41 | 35.73 |
| CES030 | 4,692,635 | 1,351,478,880 | 32.61 | 32.71 | 17.08 | 17.59 | 34.67 | 92.43 | 97.40 | 35.73 |
| CES031 | 4,566,443 | 1,315,135,584 | 32.68 | 32.82 | 17.00 | 17.50 | 34.50 | 92.02 | 97.24 | 35.66 |
| CES032 | 4,519,241 | 1,301,541,408 | 32.71 | 32.78 | 17.01 | 17.50 | 34.51 | 92.39 | 97.38 | 35.72 |
| CES033 | 4,183,416 | 1,204,823,808 | 32.70 | 32.92 | 16.94 | 17.44 | 34.38 | 92.46 | 97.42 | 35.73 |
| CES034 | 4,311,039 | 1,241,579,232 | 32.77 | 32.95 | 16.90 | 17.37 | 34.28 | 92.47 | 97.42 | 35.74 |
| CES035 | 4,262,573 | 1,227,621,024 | 32.80 | 33.01 | 16.85 | 17.34 | 34.19 | 92.10 | 97.24 | 35.66 |
| CES036 | 5,073,692 | 1,461,223,296 | 32.78 | 32.84 | 16.93 | 17.45 | 34.38 | 92.36 | 97.37 | 35.72 |
| CES037 | 4,752,831 | 1,368,815,328 | 32.86 | 32.88 | 16.86 | 17.40 | 34.26 | 92.25 | 97.32 | 35.70 |
| CES038 | 3,622,625 | 1,043,316,000 | 32.96 | 32.98 | 16.77 | 17.29 | 34.06 | 92.10 | 97.28 | 35.67 |
| CES039 | 7,486,806 | 2,156,200,128 | 32.89 | 32.93 | 16.83 | 17.34 | 34.17 | 92.33 | 97.36 | 35.71 |
| CES040 | 4,879,610 | 1,405,327,680 | 32.81 | 32.85 | 16.90 | 17.44 | 34.34 | 92.58 | 97.48 | 35.76 |

**Supplementary Table 3 (continued)**

| **Variety number** | **Read Number** | **Base Number（bp）** | **A(%)** | **T(%)** | **G(%)** | **C(%)** | **GC**  **(%)** | **Q30**  **(%)** | **Q20**  **(%)** | **Average**  **Q** |
| --- | --- | --- | --- | --- | --- | --- | --- | --- | --- | --- |
| CES041 | 3,545,143 | 1,021,001,184 | 33.03 | 33.05 | 16.70 | 17.22 | 33.91 | 89.79 | 96.20 | 35.24 |
| CES042 | 4,034,011 | 1,161,795,168 | 33.03 | 33.11 | 16.66 | 17.20 | 33.86 | 91.55 | 96.98 | 35.56 |
| CES043 | 3,752,350 | 1,080,676,800 | 33.00 | 33.12 | 16.66 | 17.22 | 33.88 | 91.40 | 96.90 | 35.53 |
| CES044 | 4,090,947 | 1,178,192,736 | 32.90 | 33.05 | 16.76 | 17.29 | 34.05 | 91.74 | 97.07 | 35.60 |
| CES045 | 4,061,645 | 1,169,753,760 | 32.82 | 32.86 | 16.89 | 17.43 | 34.33 | 91.43 | 96.93 | 35.54 |
| CES046 | 3,696,351 | 1,064,549,088 | 32.83 | 32.87 | 16.88 | 17.43 | 34.31 | 91.55 | 96.97 | 35.56 |
| CES047 | 4,224,261 | 1,216,587,168 | 32.90 | 33.00 | 16.78 | 17.32 | 34.10 | 91.57 | 96.98 | 35.57 |
| CES048 | 4,012,393 | 1,155,569,184 | 32.93 | 32.91 | 16.82 | 17.35 | 34.17 | 91.53 | 96.97 | 35.56 |
| CES049 | 3,662,831 | 1,054,895,328 | 32.78 | 32.89 | 16.91 | 17.43 | 34.33 | 91.57 | 96.99 | 35.57 |
| CES050 | 3,600,706 | 1,037,003,328 | 32.92 | 33.04 | 16.76 | 17.29 | 34.04 | 91.49 | 96.96 | 35.55 |

**Supplementary Table 4** Statistical analysis of reference genome alignment results for *C. ensifolium* genome simplification sequencing samples.

| **Variety number** | **Total**  **Reads** | **Mapped**  **Reads** | **Genome**  **Size** | **Cover**  **Size** | **Cover**  **Bases** | **Average**  **Cover depth** | **Cover**  **(5X)** | **Cover**  **(10X)** |
| --- | --- | --- | --- | --- | --- | --- | --- | --- |
| CES001 | 8,841,684 | 8,807,263(99.61%) | 3,623,198,571 | 199,267,956 | 1,268,245,872 | 6.36 | 2.00% | 0.99% |
| CES002 | 9,555,474 | 9,522,757(99.66%) | 3,623,198,571 | 187,692,674 | 1,371,277,008 | 7.31 | 2.16% | 1.16% |
| CES003 | 7,453,892 | 7,422,172(99.57%) | 3,623,198,571 | 254,969,855 | 1,068,792,768 | 4.19 | 1.55% | 0.55% |
| CES004 | 9,995,904 | 9,957,111(99.61%) | 3,623,198,571 | 242,516,785 | 1,433,823,984 | 5.91 | 2.11% | 1.05% |
| CES005 | 10,840,126 | 10,805,763(99.68%) | 3,623,198,571 | 199,811,219 | 1,556,029,872 | 7.79 | 2.23% | 1.28% |
| CES006 | 8,508,014 | 8,478,259(99.65%) | 3,623,198,571 | 234,935,400 | 1,220,869,296 | 5.2 | 1.84% | 0.85% |
| CES007 | 9,627,848 | 9,596,508(99.67%) | 3,623,198,571 | 201,219,774 | 1,381,897,152 | 6.87 | 2.03% | 1.02% |
| CES008 | 10,048,812 | 10,016,512(99.68%) | 3,623,198,571 | 182,226,546 | 1,442,377,728 | 7.92 | 2.09% | 1.17% |
| CES009 | 9,542,924 | 9,511,568(99.67%) | 3,623,198,571 | 159,630,872 | 1,369,665,792 | 8.58 | 2.00% | 1.17% |
| CES010 | 9,320,670 | 9,288,954(99.66%) | 3,623,198,571 | 271,032,338 | 1,337,609,376 | 4.94 | 1.93% | 0.86% |
| CES011 | 8,707,534 | 8,677,171(99.65%) | 3,623,198,571 | 236,619,858 | 1,249,512,624 | 5.28 | 1.90% | 0.86% |
| CES012 | 9,435,518 | 9,288,458(98.44%) | 3,623,198,571 | 226,735,319 | 1,337,537,952 | 5.9 | 1.78% | 0.75% |
| CES013 | 9,420,990 | 9,274,983(98.45%) | 3,623,198,571 | 221,598,008 | 1,335,597,552 | 6.03 | 1.77% | 0.75% |
| CES014 | 7,491,442 | 7,466,871(99.67%) | 3,623,198,571 | 211,681,168 | 1,075,229,424 | 5.08 | 1.66% | 0.71% |
| CES015 | 9,193,898 | 9,158,000(99.61%) | 3,623,198,571 | 263,132,701 | 1,318,752,000 | 5.01 | 1.95% | 0.87% |
| CES016 | 7,402,710 | 7,377,733(99.66%) | 3,623,198,571 | 251,160,609 | 1,062,393,552 | 4.23 | 1.59% | 0.59% |
| CES017 | 7,229,744 | 7,204,800(99.65%) | 3,623,198,571 | 242,169,811 | 1,037,491,200 | 4.28 | 1.57% | 0.60% |
| CES018 | 8,157,746 | 8,125,310(99.60%) | 3,623,198,571 | 206,195,005 | 1,170,044,640 | 5.67 | 1.68% | 0.68% |
| CES019 | 10,323,542 | 10,292,513(99.70%) | 3,623,198,571 | 169,439,593 | 1,482,121,872 | 8.75 | 2.06% | 1.14% |
| CES020 | 8,254,980 | 8,229,635(99.69%) | 3,623,198,571 | 215,519,271 | 1,185,067,440 | 5.5 | 1.85% | 0.85% |
| CES021 | 9,827,232 | 9,793,683(99.66%) | 3,623,198,571 | 176,456,181 | 1,410,290,352 | 7.99 | 2.13% | 1.24% |
| CES022 | 10,401,838 | 10,367,677(99.67%) | 3,623,198,571 | 187,658,575 | 1,492,945,488 | 7.96 | 2.20% | 1.26% |
| CES023 | 11,627,778 | 11,586,127(99.64%) | 3,623,198,571 | 186,579,342 | 1,668,402,288 | 8.94 | 2.31% | 1.40% |
| CES024 | 8,370,366 | 8,341,812(99.66%) | 3,623,198,571 | 213,202,457 | 1,201,220,928 | 5.63 | 1.81% | 0.84% |
| CES025 | 8,866,440 | 8,835,215(99.65%) | 3,623,198,571 | 209,505,947 | 1,272,270,960 | 6.07 | 1.96% | 0.97% |
| CES026 | 10,571,832 | 10,535,173(99.65%) | 3,623,198,571 | 195,567,850 | 1,517,064,912 | 7.76 | 2.21% | 1.23% |
| CES027 | 10,424,366 | 10,390,792(99.68%) | 3,623,198,571 | 193,830,598 | 1,496,274,048 | 7.72 | 2.22% | 1.28% |
| CES028 | 9,490,942 | 9,454,881(99.62%) | 3,623,198,571 | 227,878,619 | 1,361,502,864 | 5.97 | 2.05% | 1.03% |
| CES029 | 9,614,478 | 9,583,450(99.68%) | 3,623,198,571 | 177,905,217 | 1,380,016,800 | 7.76 | 2.11% | 1.13% |
| CES030 | 9,385,270 | 9,342,439(99.54%) | 3,623,198,571 | 182,775,690 | 1,345,311,216 | 7.36 | 2.09% | 1.09% |
| CES031 | 9,132,886 | 9,102,108(99.66%) | 3,623,198,571 | 180,377,193 | 1,310,703,552 | 7.27 | 1.99% | 1.03% |
| CES032 | 9,038,482 | 8,995,563(99.53%) | 3,623,198,571 | 226,872,066 | 1,295,361,072 | 5.71 | 2.01% | 0.89% |
| CES033 | 8,366,832 | 8,332,513(99.59%) | 3,623,198,571 | 229,593,905 | 1,199,881,872 | 5.23 | 1.77% | 0.77% |
| CES034 | 8,622,078 | 8,593,064(99.66%) | 3,623,198,571 | 234,634,374 | 1,237,401,216 | 5.27 | 1.87% | 0.84% |
| CES035 | 8,525,146 | 8,381,377(98.31%) | 3,623,198,571 | 229,288,596 | 1,206,918,288 | 5.26 | 1.60% | 0.62% |
| CES036 | 10,147,384 | 10,111,847(99.65%) | 3,623,198,571 | 175,420,776 | 1,456,105,968 | 8.3 | 2.14% | 1.24% |
| CES037 | 9,505,662 | 9,473,990(99.67%) | 3,623,198,571 | 167,923,525 | 1,364,254,560 | 8.12 | 2.07% | 1.19% |
| CES038 | 7,245,250 | 7,219,998(99.65%) | 3,623,198,571 | 144,902,046 | 1,039,679,712 | 7.18 | 1.74% | 0.84% |
| CES039 | 14,973,612 | 14,915,568(99.61%) | 3,623,198,571 | 198,428,923 | 2,147,841,792 | 10.82 | 2.59% | 1.73% |

**Supplementary Table 4 (continued)**

| **Variety number** | **Total**  **Reads** | **Mapped**  **Reads** | **Genome**  **Size** | **Cover**  **Size** | **Cover**  **Bases** | **Average**  **Cover depth** | **Cover**  **(5X)** | **Cover**  **(10X)** |
| --- | --- | --- | --- | --- | --- | --- | --- | --- |
| CES040 | 9,759,220 | 9,725,622(99.66%) | 3,623,198,571 | 169,996,842 | 1,400,489,568 | 8.24 | 2.09% | 1.20% |
| CES041 | 7,090,286 | 6,996,282(98.67%) | 3,623,198,571 | 184,966,018 | 1,007,464,608 | 5.45 | 1.68% | 0.64% |
| CES042 | 8,068,022 | 8,001,801(99.18%) | 3,623,198,571 | 189,334,786 | 1,152,259,344 | 6.09 | 1.89% | 0.78% |
| CES043 | 7,504,700 | 7,395,367(98.54%) | 3,623,198,571 | 162,543,700 | 1,064,932,848 | 6.55 | 1.48% | 0.60% |
| CES044 | 8,181,894 | 8,116,330(99.20%) | 3,623,198,571 | 196,683,408 | 1,168,751,520 | 5.94 | 1.81% | 0.72% |
| CES045 | 8,123,290 | 8,095,995(99.66%) | 3,623,198,571 | 168,444,033 | 1,165,823,280 | 6.92 | 1.85% | 0.92% |
| CES046 | 7,392,702 | 7,367,508(99.66%) | 3,623,198,571 | 147,669,616 | 1,060,921,152 | 7.18 | 1.70% | 0.81% |
| CES047 | 8,448,522 | 8,381,669(99.21%) | 3,623,198,571 | 190,978,728 | 1,206,960,336 | 6.32 | 1.89% | 0.78% |
| CES048 | 8,024,786 | 7,997,590(99.66%) | 3,623,198,571 | 161,682,494 | 1,151,652,960 | 7.12 | 1.89% | 0.98% |
| CES049 | 7,325,662 | 7,300,538(99.66%) | 3,623,198,571 | 163,173,839 | 1,051,277,472 | 6.44 | 1.70% | 0.78% |
| CES050 | 7,201,412 | 7,172,971(99.61%) | 3,623,198,571 | 155,273,596 | 1,032,907,824 | 6.65 | 1.64% | 0.80% |

**Supplementary Table 5** Statistical analysis of the detected SNP information.

| **Variety number** | **SNP Number** | **Transition** | **Transversion** | **Ti/Tv** | **Heterozygosity** | **Homozygosity** |
| --- | --- | --- | --- | --- | --- | --- |
| CES001 | 890,790 | 594,981 | 295,809 | 2.011369 | 479,343 | 411,447 |
| CES002 | 941,045 | 627,339 | 313,706 | 1.999767 | 506,141 | 434,904 |
| CES003 | 748,040 | 497,668 | 250,372 | 1.987714 | 299,619 | 448,421 |
| CES004 | 874,106 | 582,460 | 291,646 | 1.997147 | 422,879 | 451,227 |
| CES005 | 978,158 | 655,802 | 322,356 | 2.034403 | 525,715 | 452,443 |
| CES006 | 743,877 | 496,489 | 247,388 | 2.006924 | 249,584 | 494,293 |
| CES007 | 980,737 | 654,408 | 326,329 | 2.005363 | 453,947 | 526,790 |
| CES008 | 883,812 | 589,300 | 294,512 | 2.000937 | 443,547 | 440,265 |
| CES009 | 863,937 | 579,696 | 284,241 | 2.039452 | 319,355 | 544,582 |
| CES010 | 721,982 | 480,976 | 241,006 | 1.995701 | 287,314 | 434,668 |
| CES011 | 925,389 | 617,573 | 307,816 | 2.006306 | 464,439 | 460,950 |
| CES012 | 1,364,127 | 854,786 | 509,341 | 1.67822 | 311,016 | 1,053,111 |
| CES013 | 1,363,736 | 855,886 | 507,850 | 1.685313 | 311,818 | 1,051,918 |
| CES014 | 834,738 | 558,355 | 276,383 | 2.020222 | 350,221 | 484,517 |
| CES015 | 886,978 | 593,686 | 293,292 | 2.024215 | 436,128 | 450,850 |
| CES016 | 691,307 | 463,893 | 227,414 | 2.039861 | 319,053 | 372,254 |
| CES017 | 709,732 | 471,978 | 237,754 | 1.985153 | 324,704 | 385,028 |
| CES018 | 1,612,362 | 1,063,302 | 549,060 | 1.936586 | 724,097 | 888,265 |
| CES019 | 867,574 | 579,496 | 288,078 | 2.011594 | 470,537 | 397,037 |
| CES020 | 811,758 | 542,843 | 268,915 | 2.018642 | 371,209 | 440,549 |
| CES021 | 857,411 | 574,496 | 282,915 | 2.030631 | 392,583 | 464,828 |
| CES022 | 897,307 | 600,946 | 296,361 | 2.02775 | 446,443 | 450,864 |
| CES023 | 1,008,720 | 675,601 | 333,119 | 2.028107 | 449,222 | 559,498 |
| CES024 | 773,076 | 514,791 | 258,285 | 1.993112 | 354,214 | 418,862 |
| CES025 | 941,763 | 628,863 | 312,900 | 2.009789 | 418,389 | 523,374 |
| CES026 | 948,023 | 634,702 | 313,321 | 2.025724 | 471,868 | 476,155 |
| CES027 | 895,664 | 599,599 | 296,065 | 2.025228 | 450,779 | 444,885 |
| CES028 | 883,803 | 591,383 | 292,420 | 2.022375 | 425,628 | 458,175 |
| CES029 | 929,367 | 621,212 | 308,155 | 2.015908 | 463,093 | 466,274 |
| CES030 | 944,256 | 632,167 | 312,089 | 2.025598 | 463,172 | 481,084 |
| CES031 | 890,582 | 594,568 | 296,014 | 2.008581 | 445,521 | 445,061 |
| CES032 | 1,517,733 | 1,018,838 | 498,895 | 2.042189 | 707,926 | 809,807 |
| CES033 | 811,794 | 540,406 | 271,388 | 1.991267 | 302,243 | 509,551 |
| CES034 | 786,274 | 523,467 | 262,807 | 1.991831 | 378,644 | 407,630 |
| CES035 | 1,261,906 | 788,620 | 473,286 | 1.666265 | 281,758 | 980,148 |
| CES036 | 938,219 | 629,277 | 308,942 | 2.036877 | 384,361 | 553,858 |
| CES037 | 937,657 | 628,083 | 309,574 | 2.028862 | 422,842 | 514,815 |
| CES038 | 795,650 | 530,865 | 264,785 | 2.004891 | 388,516 | 407,134 |
| CES039 | 1,067,435 | 715,886 | 351,549 | 2.036376 | 450,007 | 617,428 |
| CES040 | 933,946 | 626,334 | 307,612 | 2.036117 | 381,971 | 551,975 |
| CES041 | 1,170,769 | 747,231 | 423,538 | 1.76426 | 470,026 | 700,743 |
| CES042 | 1,245,912 | 796,143 | 449,769 | 1.770115 | 497,648 | 748,264 |

**Supplementary Table 5 (continued)**

| **Variety number** | **SNP Number** | **Transition** | **Transversion** | **Ti/Tv** | **Heterozygosity** | **Homozygosity** |
| --- | --- | --- | --- | --- | --- | --- |
| CES043 | 1,289,853 | 806,199 | 483,654 | 1.666892 | 299,752 | 990,101 |
| CES044 | 1,254,157 | 800,175 | 453,982 | 1.76257 | 496,348 | 757,809 |
| CES045 | 905,503 | 605,260 | 300,243 | 2.0159 | 425,213 | 480,290 |
| CES046 | 861,602 | 576,960 | 284,642 | 2.026967 | 425,289 | 436,313 |
| CES047 | 1,301,996 | 831,564 | 470,432 | 1.76766 | 517,232 | 784,764 |
| CES048 | 784,842 | 524,493 | 260,349 | 2.014577 | 423,510 | 361,332 |
| CES049 | 881,929 | 589,171 | 292,758 | 2.012485 | 425,314 | 456,615 |
| CES050 | 796,603 | 529,069 | 267,534 | 1.977577 | 307,031 | 489,572 |
